# Enhanced prediction of bolt support drilling pressure using optimized Gaussian process regression

**DOI:** 10.1038/s41598-024-52420-w

**Published:** 2024-01-26

**Authors:** Jie Liu

**Affiliations:** 1 CCTEG Taiyuan Research Institute Co., Ltd., Taiyuan, 030000 China; 2 Shanxi Tiandi Coal Mining Machinery Co., Ltd., Taiyuan, 030000 China; 3 China National Engineering Laboratory for Coal Mining Machinery, Taiyuan, 030000 China

**Keywords:** Mechanical engineering, Information technology, Scientific data

## Abstract

This study introduces a novel method for predicting drilling pressure in bolt support systems by optimizing Gaussian process time series regression (GPR) using hybrid optimization algorithms. The research initially identified significant variations in prediction outcomes based on different kernel functions and historical points combinations in the GPR algorithm. To address this, we explored 160 distinct schemes combining 10 kernel functions and 16 historical points for numerical analysis. Applying three hybrid optimization algorithms—Genetic Algorithm-GPR (GA-GPR), Particle Swarm Optimization-GPR (PSO-GPR), and Ant Colony Algorithm-GPR (ACA-GPR)—we iteratively optimized these key parameters. The PSO-GPR algorithm emerged as the most effective, achieving an 80% prediction accuracy with a deviation range of 1–2 MPa, acceptable in practical drilling operations. This optimization led to the RQ kernel function with 18 historical points as the optimal combination, yielding an RMSE value of 0.0047246, in contrast to the least effective combination (E kernel function with 6 historical points) producing an RMSE of 0.035704. The final outcome of this study is a robust and efficient prediction system for underground bolt support drilling pressure, verified through practical application. This approach significantly enhances the accuracy and efficiency of support systems in geotechnical engineering, demonstrating the practical applicability of the PSO-GPR model in real-world scenarios.

## Introduction

Throughout the entire process of underground bolt support, the unpredictability of drilling pressure has become a critical factor limiting support efficiency. Given the non-linear changes in the hardness of coal and rock geology, the drilling angle and feed rate of the drilling rig must be continually adjusted through human intervention during drill pipe drilling. This adjustment aims to balance the drill rig's working power and load, albeit with considerable drawbacks including extended adjustment times, reduced precision, operational lags, and significant potential safety risks. Furthermore, adaptive drilling control based on sensor detection invariably incurs a delay. This control mode requires intervention post-drilling, and the control logic cannot be preplanned for the entirety of the next drilling process. Therefore, accurately predicting the surrounding rock's drilling pressure is the key challenge to achieving intelligent automatic safety support.

In earlier studies by numerous researchers, the pre-drilling pressure was primarily predicted using the seismic interval velocity method. Scholars such as Khattab used high-resolution full waveform inversion velocity from 1D seismic velocity profiles to 3D modeling to predict pre-drilling pore pressure^1^. Noah and others used interval seismic velocity to predict the pre-drilling pore pressure of land oilfields^2^, while Ayodele and colleagues used tomography generated by two-dimensional seismic data to extract grid maps to delineate pre-drilling overpressure and normal pressure formation pore pressure^3^. This method is regional, requiring consideration of multiple factors such as formation origin, rock type and structure, abrupt geological boundaries, and faults. However, its generalization ability is relatively weak, making it unsuitable for predicting bolt support's pre-drilling pressure. Some researchers, like Haris et al., have attempted to use machine learning methods to predict pre-drilling pressure, such as employing probabilistic neural networks to predict pore pressure in the South Sumatra Basin^4^. However, no corresponding research exists for pre-drilling pressure prediction in underground bolt support.

In the entire process of roadway excavation and support, the closer the support position, the higher the probability of increased hardness in the surrounding rock. Within the same roadway, the hardness of the surrounding rock at a certain distance may be similar, or there may be local mutations, with its changes being non-linear. The Gaussian process time series regression algorithm demonstrates good adaptability to the time series data analysis of drilling pressure in the process of bolt support.

Gaussian process time series regression is a machine learning method based on Gaussian random processes, kernel functions, Bayesian theory, and time series data input. Compared to other machine learning methods, Gaussian process time series regression is less challenging to implement, can adaptively obtain hyperparameters, flexibly infer non-parameters, and the output value has probabilistic significance. The study of Gaussian process regression originated in the 1940s and was used in signal filtering technology^5^. In recent years, an increasing number of researchers have begun to apply it to practical engineering applications, especially in the fields of astronomy^6^, civil engineering^7^, battery chemistry^8^, and others.

Scholars have contributed significantly to the field of Gaussian process regression in various ways. Yang et al. proposed a deep Gaussian process regression (DGPR) method for evaluating the State of Health (SOH) of lithium-ion batteries, leveraging both Gaussian processes and deep networks^9^. Hong et al. expanded the applicability of the Iterative Power and Amplitude Correction (IPAC) algorithm to simulate non-stationary and non-Gaussian processes, considering five transformation pairs^10^. Zhang et al. successfully reduced computational complexity by exploiting the Kronecker structure presented in the state-space model of spatiotemporal Gaussian processes, verifying their findings through the application of weather data prediction^11^. In the realm of 3D surface modeling, Zhao et al. proposed a spherical multi-output Gaussian process method^12^. Shadab et al. offered a systematic method based on the black box model and experimental design to establish an alternative model for predicting and estimating transformer top oil temperature parameters^13^. Gao et al. introduced a residual fatigue life prediction method for metal materials based on Gaussian process regression, which is employed to predict the residual fatigue life of metal materials under two-step loading^14^. Meanwhile, Zeng et al. utilized Gaussian process regression to predict building power consumption^15^. Jo et al. proposed a machine learning framework for path loss modeling that is based on multi-dimensional regression of artificial neural networks (ANN), variance analysis via Gaussian processes, and feature selection assisted by Principal Component Analysis (PCA)^16^. Lastly, Rong et al. developed a data-driven nonparametric Bayesian model based on Gaussian processes to describe and predict in real time the uncertainties in ship lateral motion and trajectory^17^.

Recent advancements in Gaussian process regression (GPR) have seen its application extend across various domains of geotechnical engineering. For instance, a study applied GPR to predict geological parameters, such as Rock Quality Designation (RQD) along tunnel routes, demonstrating GPR's utility in underground engineering^18^. Similarly, Gaussian process regression has been explored for predicting the California bearing ratio of HARHA-treated expansive soils, highlighting the versatility of GPR in soil mechanics^19^. Additionally, GPR has been employed to estimate the trend and random components of soil properties at different locations, indicating its potential for spatial analysis in geotechnical contexts^20^. Another significant application includes the use of GPR for effective regional post-earthquake building damage inference, showcasing its proficiency in structural damage assessment^21^. Furthermore, the integration of GPR in Monte Carlo simulations for the stability analysis of slopes with varied soil strength underlines its value in risk assessment and safety evaluation in geotechnical engineering^22^. These studies underscore the growing importance and diverse applicability of GPR in geotechnical engineering, yet also indicate the necessity for further exploration, particularly in the area of bolt support drilling pressure prediction, which this study addresses.

The selection of key parameters, such as the kernel function and historical points, in Gaussian process time series regression remains under-explored in geotechnical engineering applications. Existing research often relies on conventional choices like the Squared Exponential (SE) kernel function, with parameters typically based on heuristic methods rather than systematic analysis. This gap highlights a need for a more systematic approach to determine how these critical parameters influence the model's predictive accuracy and reliability in the context of bolt support drilling pressure.

In this study, we propose a refined method for predicting bolt support drilling pressure in underground settings, employing a hybrid optimization algorithm to precisely determine the key parameters in Gaussian process time series regression. Our initial step involves modeling the Gaussian process time series regression algorithm, where we examine the impact of various kernel functions and historical points on prediction outcomes. This is achieved by analyzing 160 different combinations of 10 kernel functions and 16 historical points against underground bolt support drilling pressure data. Subsequently, we utilize genetic, particle swarm, and ant colony optimization algorithms to iteratively refine these parameters. This methodology not only identifies the most effective parameter combinations but also leads to the development of a robust and reliable prediction system for bolt support drilling pressure in underground environments. We further validate the system's practical applicability and generalization capacity by applying the optimized parameters in diverse underground drilling scenarios.

This study introduces a novel approach to predict drilling pressure in bolt support systems, leveraging machine learning techniques. Unlike traditional methods such as the seismic interval velocity method, which predominantly rely on material analysis and have shown limitations in generalization and adaptability, our approach uses a hybrid algorithm to optimize Gaussian Process Time Series Regression parameters. This method not only addresses the gaps in existing literature by providing a more versatile and accurate tool for predicting drilling pressure but also represents the first known application of machine learning for this specific purpose in underground bolt support systems. By combining the strengths of various optimization algorithms (GA-GPR, PSO-GPR, ACA-GPR) and comparing their efficacy, this research offers significant insights into the potential of machine learning in enhancing the predictability and safety of underground drilling operations.

## Gaussian process time seriesregression algorithm

### Algorithmic modeling

Building on the established theoretical framework of Gaussian Process Regression (GPR), our approach utilizes this advanced statistical technique to model and analyze drilling pressure data. GPR, a non-parametric, Bayesian approach to regression, offers flexibility and precision, making it ideal for complex datasets like those in geotechnical engineering. The foundational concepts and mathematical formulations of GPR are extensively covered in the seminal work by Rasmussen and Williams^23^. The following Eqs. (1–14) are derived based on their methodology.

Suppose that the sample training set composed of drilling pressure data in the process of roadway excavation is:

1 $${\varvec{Q}} = \left\{ {({\varvec{x}}_{i} ,y_{i} )\;\left| {\;i = 1, \ldots n} \right.} \right\} = \left\{ {{\varvec{X}},{\varvec{y}}} \right\}$$

Consider a sample training set composed of drilling pressure data from the roadway excavation process. Here, ${\varvec{x}}_{i}$ represents the sample input vector of test data, and $y_{i}$ is the output value. The drilling pressure training input vector matrix and the output values are denoted by ${\varvec{X}}$ and ${\varvec{y}}$ vectors respectively. Therefore, the output vector can be expressed as:

2 $${\varvec{y}} = {\varvec{x}}^{{\text{T}}} {\varvec{w}} + {{\user2{\upvarepsilon}}}$$

In this equation, ${\varvec{w}}$ is the weight vector, assumed to follow a Gaussian distribution ${\varvec{w}}\sim\;N({\varvec{0}},{{\user2{\Sigma}}}_{p} )$, and ${{\user2{\upvarepsilon}}}$ denotes noise, adhering to the standard Gaussian distribution $\varepsilon \sim\;N(0,\sigma_{n}^{2} )$. According to Bayesian theory, the posterior distribution of weight ${\varvec{w}}$ is represented as:

3 $$p({\varvec{w}}\;\left| {\;{\varvec{y,X}}} \right.) = \frac{{p({\varvec{w}})p({\varvec{y}}\;\left| {{\varvec{X,w}}} \right.)}}{{p({\varvec{y}}\;\left| {\;{\varvec{X}}} \right.)}}$$

The likelihood $p({\varvec{y}}\;\left| {\;{\varvec{X,w}}} \right.)$ is expressed as:

4 $$\begin{aligned} p({\varvec{y}}\;\left| {\;{\varvec{X,w}}} \right.) & = \prod\limits_{i = 1}^{n} {p(y_{i} \;\left| {\;{\varvec{x}}_{i} } \right.,{\varvec{w}})} \\ & = \prod\limits_{i = 1}^{n} {\frac{1}{{\sqrt {2\pi } \sigma_{n} }}} \exp \left( { - \frac{{(y_{i} - {\varvec{x}}_{i}^{{\text{T}}} {\varvec{w}})^{2} }}{{2\sigma_{n}^{2} }}} \right) \\ & = \frac{1}{{(2\pi \sigma_{n}^{2} )^{n/2} }}\exp \left( { - \frac{1}{{2\sigma_{n}^{2} }}\left\| {\;{\varvec{y}} - {\varvec{X}}^{{\varvec{T}}} {\varvec{w}}\;} \right\|^{2} } \right) \\ & = N({\varvec{X}}^{{\text{T}}} {\varvec{w}},\sigma_{n}^{2} {\varvec{I}}) \\ \end{aligned}$$

The marginal likelihood in formula(3), $p({\varvec{y}}\;\left| {\;{\varvec{X}}} \right.)$, is expressed as:

5 $$p({\varvec{y}}\;\left| {\;{\varvec{X}}} \right.) = \int {p({\varvec{y}}\;\left| {\;{\varvec{X,w}}} \right.)} p({\varvec{w}}){\text{d}}{\varvec{w}}$$

As the marginal likelihood is independent of weight, we can apply the Gaussian distribution's conditional probability formula and incorporate formulae (3), (4) and (5) to obtain the posterior distribution of weight ${\varvec{w}}$. It reveals that the weight ${\varvec{w}}$'s posterior distribution follows the Gaussian distribution $p({\varvec{w}}\;\left| {\;{\varvec{y,X}}} \right.)\sim\;N(\overline{{\varvec{w}}} ,{\varvec{A}}^{ - 1} )$, where $\overline{{\varvec{w}}} = \frac{1}{{\sigma_{n}^{2} }}{\varvec{A}}^{ - 1} {\varvec{Xy}}$, ${\varvec{A}} = \frac{1}{{\sigma_{n}^{2} }}{\varvec{XX}}^{{\text{T}}} + {{\user2{\Sigma}}}_{p}^{ - 1}$.

6 $$p({\varvec{w}}\;\left| {\;{\varvec{y,X}}} \right.) \propto {\text{exp}}\left( { - \frac{1}{2}({\varvec{w}} - \overline{{\varvec{w}}} )^{{\text{T}}} \left( {\frac{1}{{\sigma_{n}^{2} }}{\varvec{XX}}^{{\text{T}}} + {{\user2{\Sigma}}}_{p}^{ - 1} } \right)({\varvec{w}} - \overline{{\varvec{w}}} )} \right)$$

7 $$\overline{{\varvec{w}}} = \sigma_{n}^{ - 2} (\sigma_{n}^{ - 2} {\varvec{XX}}^{{\text{T}}} + {{\user2{\Sigma}}}_{p}^{ - 1} )^{ - 1} {\varvec{Xy}}$$

The weight ${\varvec{w}}$ is then calculated by marginalization, leading to the posterior distribution of the function $f_{*}$ corresponding to the new input sample matrix ${\varvec{x}}_{*}$:

8 $$\begin{aligned} p(f_{*} \;\left| {\;{\varvec{x}}_{*} } \right.{\varvec{,X,y}}) & = \int {p(f_{*} \;\left| \; \right.{\varvec{x}}_{*} ,{\varvec{w}})} p({\varvec{w}}\;\left| {\;{\varvec{X,y}}} \right.){\text{d}}{\varvec{w}} \\ & = N\left( {\frac{1}{{\sigma_{n}^{2} }}{\varvec{x}}_{*}^{{\text{T}}} {\varvec{A}}^{ - 1} {\varvec{Xy}},{\varvec{x}}_{*}^{{\text{T}}} {\varvec{A}}^{ - 1} {\varvec{x}}_{*} } \right) \\ \end{aligned}$$

When handling nonlinear data problems, a mapping function can be employed to map the input vector ${\varvec{x}}$ into an $N$ dimensional feature space. Here, the training data ${\varvec{x}}$ corresponds to the input vector ${\user2{\varPhi}}({\varvec{x}})$, and the training data set ${\varvec{X}}$ (which includes the training data) corresponds to the input vector matrix ${{\user2{\Phi}}}({\varvec{X}}) = \left\{ {\;{\varvec{\varphi }}^{(1)} ({\varvec{x}}),{\varvec{\varphi }}^{(2)} ({\varvec{x}}) \cdots {\varvec{\varphi }}^{(n)} ({\varvec{x}})\;} \right\}_{N \times n}$. The new input sample ${\varvec{x}}_{*}$ is associated with the new input sample vector ${\varvec{\varphi }}({\varvec{x}}_{*} )$, and the new input sample set ${\varvec{X}}_{*}$ (which includes the test data) corresponds to the new input sample matrix ${{\user2{\Phi}}}({\varvec{X}}_{*} ) = \left\{ {\;{\varvec{\varphi }}^{(1)} ({\varvec{x}}_{*} ),{\varvec{\varphi }}^{(2)} ({\varvec{x}}_{*} ) \cdots {\varvec{\varphi }}^{(m)} ({\varvec{x}}_{*} )\;} \right\}_{N \times m}$.

The prediction distribution for nonlinear data problems can then be obtained through:

9 $$f_{*} \;\left| {\;{\varvec{x}}} \right._{*} ,{\varvec{X}},{\varvec{y}}\sim\;N\left( \begin{gathered} \frac{1}{{\sigma_{n}^{2} }}{\varvec{\varphi }}({\varvec{x}}_{*} )^{{\text{T}}} {\varvec{A}}^{ - 1} {{\user2{\Phi}}}({\varvec{X}}){\varvec{y}}, \hfill \\ {\varvec{\varphi }}({\varvec{x}}_{*} )^{{\text{T}}} {\varvec{A}}^{ - 1} {\varvec{\varphi }}({\varvec{x}}_{*} ) \hfill \\ \end{gathered} \right)$$

By substitifying ${{\user2{\Phi}}} = {{\user2{\Phi}}}({\varvec{X}})$, ${\varvec{\varphi }}_{*} = {\varvec{\varphi }}({\varvec{x}}_{*} )$, ${\varvec{K}} = {{\user2{\Phi}}}^{{\text{T}}} {{\user2{\Sigma}}}_{p} {{\user2{\Phi}}}$,we can obtain:

10 $$f_{*} \;\left| {\;{\varvec{x}}_{*} } \right.,{\varvec{X}},{\varvec{y}}\sim\;N\left( \begin{gathered} {\varvec{\varphi }}_{*}^{{\text{T}}} {{\user2{\Sigma}}}_{p} {{\user2{\Phi}}}({\varvec{K}} + \sigma_{n}^{2} {\varvec{I}})^{ - 1} {\varvec{y}}, \hfill \\ {\varvec{\varphi }}_{*}^{{\text{T}}} {{\user2{\Sigma}}}_{p} {\varvec{\varphi }}_{*} - {\varvec{\varphi }}_{*}^{{\text{T}}} {{\user2{\Sigma}}}_{p} {{\user2{\Phi}}}({\varvec{K}} + \sigma_{n}^{2} {\varvec{I}})^{ - 1} {{\user2{\Phi}}}^{{\text{T}}} {{\user2{\Sigma}}}_{p} {\varvec{\varphi }}_{*} \hfill \\ \end{gathered} \right)$$

To introduce kernel functions, we propose:

11 $$k({\varvec{x}},{\varvec{x^{\prime}}}) = {\varvec{\varphi }}({\varvec{x}})^{{\text{T}}} {{\user2{\Sigma}}}_{p} {\varvec{\varphi }}({\varvec{x^{\prime}}})$$

where ${{\user2{\Sigma}}}_{p}$ is a covariance matrix of order N, which is a real symmetric positive semi-definite matrix. Thus, there is an orthogonal matrix ${\varvec{U}}$ that satisfies ${{\user2{\Sigma}}}_{p} = {\varvec{U\Lambda U}}^{{\text{T}}}$, where ${\varvec{U}}$ is an orthogonal matrix composed of ${{\user2{\Sigma}}}_{p}$'s eigenvector columns, and ${{\user2{\Lambda}}}$ is a diagonal matrix composed of ${{\user2{\Sigma}}}_{p}$'s eigenvalues. Hence:

12 $$({{\user2{\Sigma}}}_{p}^{1/2} )^{2} = {\varvec{U\Lambda }}^{{1/2}} {\varvec{U}}^{{\text{T}}} {\varvec{U\Lambda }}^{{1/2}} {\varvec{U}}^{{\text{T}}}$$

Simultaneously solving the above equation with formula (12), we can transform the kernel function (11) into:

13 $$k({\varvec{x,x^{\prime}}}) = {\varvec{\varphi }}({\varvec{x}})^{{\text{T}}} {\varvec{\varphi }}({\varvec{x^{\prime}}})$$

The prediction distribution of the nonlinear data problem based on the kernel function matrix is:

14 $$f_{*} \;\left| {\;{\varvec{X}}} \right._{*} ,{\varvec{X}},{\varvec{y}}\sim\;N\left( \begin{gathered} {\varvec{K}}({\varvec{X}}_{*} ,{\varvec{X}})\left[ {{\varvec{K}}({\varvec{X}},{\varvec{X}}) + \sigma_{n}^{2} {\varvec{I}}} \right]^{ - 1} {\varvec{y}}, \hfill \\ {\varvec{K}}({\varvec{X}}_{*} ,{\varvec{X}}_{*} ) - {\varvec{K}}({\varvec{X}}_{*} ,{\varvec{X}})\left[ {{\varvec{K}}({\varvec{X}},{\varvec{X}}) + \sigma_{n}^{2} {\varvec{I}}} \right]^{ - 1} {\varvec{K}}^{{\text{T}}} ({\varvec{X}}_{*} ,{\varvec{X}}) \hfill \\ \end{gathered} \right)$$

Here, ${\varvec{K}}({\varvec{X}},{\varvec{X}})$ is the kernel function matrix between the input training data, ${\varvec{K}}({\varvec{X}}_{*} ,{\varvec{X}})$ is the kernel function matrix between the input test data and input training data, and ${\varvec{K}}({\varvec{X}}_{*} ,{\varvec{X}}_{*} )$ is the kernel function matrix between the input test data.

### Algorithm evaluation Indicators

The algorithm's application effect can be quantitatively analyzed by assessing the solution's impact – that is, the predictive effect. Commonly used evaluation indices include the coefficient of determination (R2), mean square error (MSE), root mean square error (RMSE), mean absolute error (MAE), mean relative error (MRE), and the variance of prediction error (PEV), among others.

In this study, due to the large number of samples, we need to consider and evaluate the overall system's solution effect, eliminate the influence of individual maxima or minima on system prediction, and objectively explain the overall prediction performance. This aligns with the evaluation characteristics of RMSE as described in James et al.^24^.

The RMSE evaluation formula is:

15 $${\text{RMSE}} = \sqrt {\frac{{\sum\nolimits_{i = 1}^{n} {(\hat{y}_{i} - y_{i} )} }}{n}}$$

For the RMSE index, its value range is 0 ~ ∞. The lower the value, the better the evaluation.

### Influence of kernel function and historical points

#### Kernel function and hyper-parameter determination

In order to achieve optimum training and prediction results within the Gaussian process for time-series regression, it is vital to select the right kernel function and determine the corresponding hyperparameters.

Kernel functions are primarily divided into two categories: isotropic (ISO) kernel functions and automatic relevance determination (ARD) kernel functions. The hyperparameter in the ISO kernel is a one-dimensional scalar, whereas the hyperparameter in the ARD kernel is a multi-dimensional vector, corresponding to the number of input vectors. Therefore, the ARD kernel implies more computational effort, but it also enables the removal of unrelated inputs. In this study, we selected five classical kernel functions from these categories for screening: E, SE, RQ, Matern3/2, Matern5/2 for ISO kernel functions, and ARDE, ARDSE, ARDRQ, ARDMatern3/2, ARDMatern5/2 for ARD kernel functions.

The mathematical formulation and effectiveness of these kernel functions within Gaussian processes have been comprehensively discussed in the work of Schölkopf and Smola. Similarly, the process of hyperparameter determination, especially the computation of the negative log marginal likelihood (Formula 17), is a critical aspect of optimizing the Gaussian process model, as detailed in the same reference^25^.

Taking the Matern3/2 kernel function as an example, its expression involves the sum of unknown parameters $\sigma_{f}$ and $\sigma_{l}$, which are considered hyperparameters.

16 $$k({\varvec{x}}_{i} ,{\varvec{x}}_{j} ) = \sigma_{f}^{2} \left( {1 + \frac{{\sqrt 3 \left\| {\;{\varvec{x}}_{i} - {\varvec{x}}_{j} \;} \right\|}}{{\sigma_{l} }}} \right)\exp \left( { - \frac{{\sqrt 3 \left\| {\;{\varvec{x}}_{i} - {\varvec{x}}_{j} \;} \right\|}}{{\sigma_{l} }}} \right)$$

The optimal values are obtained using the method of maximum marginal likelihood. The marginal likelihood function for the input training data set is then the negative logarithm of the marginal likelihood.

17 $$\begin{aligned} - \log p({\varvec{y}}\;\left| {\;{\varvec{X,\theta }}} \right.) & = \frac{1}{2}{\varvec{y}}^{{\text{T}}} ({\varvec{K}} + \sigma_{n}^{2} {\varvec{I}})^{ - 1} {\varvec{y}} \\ & + \frac{1}{2}{\text{log}}\left| {\;{\varvec{K}} + \sigma_{n}^{2} {\varvec{I}}\;} \right| + \frac{n}{2}\log 2\pi \\ \end{aligned}$$

This function serves as the minimization objective function to search for optimal hyperparameters.

#### Historical points

Gaussian process time-series regression employs a Gaussian process regression algorithm for time-series data processing. For instance, as time progresses and roadway excavation continues, the drilling pressure value for each supporting bolt can be used to form the input vector for Gaussian process time-series regression.

Assume that during the roadway excavation and support process, the time-series sample vector created by the drilling pressure of the same row of anchor rods is $\left\{ {x_{1} ,x_{2} \ldots x_{n} } \right\}$. The first *h* group data forms the input vector of the training set, and the *h* + 1 data acts as the output value of the training set, thus forming the first training sample pair. Then, using the second data to the *h* + 1 data as the input vector of the training set, and the *h* + 2 data as the output value of the training set, the second training sample pair is formed, and so on. The number of historical points is crucial to the performance of the algorithm, affecting the number of training sample pairs and the global changes considered by the training samples.

#### Impact of key parameters on results

We collected drilling pressure test data (Supplementary Information 1) during the excavation process of a roadway, specifically when drilling 1000 mm. The dataset comprised a total of 1000 samples, partitioned into 700 training samples and 300 test samples. We considered 10 kernel functions (E, SE, RQ, Matern3/2, Matern5/2, ARDE, ARDSE, ARDRQ, ARDMatern3/2, ARDMatern5/2) and 16 historical points (5–20), which were combined into 160 distinct schemes for numerical analysis.

**Figure 1:**
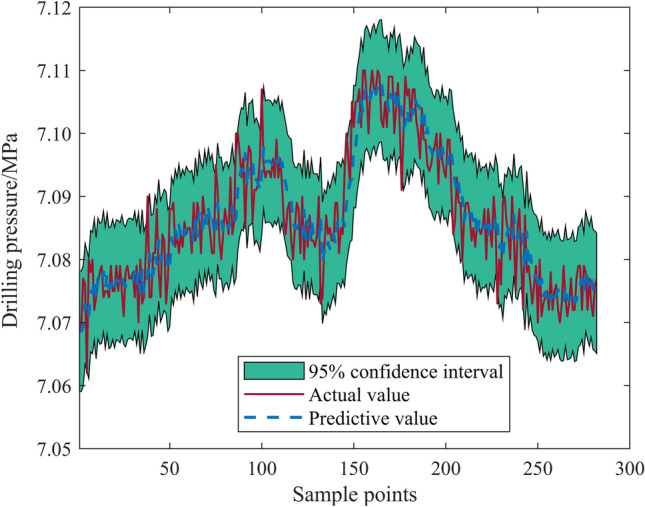
Test set solution results (kernel function = RQ, historical points = 18).

**Figure 2:**
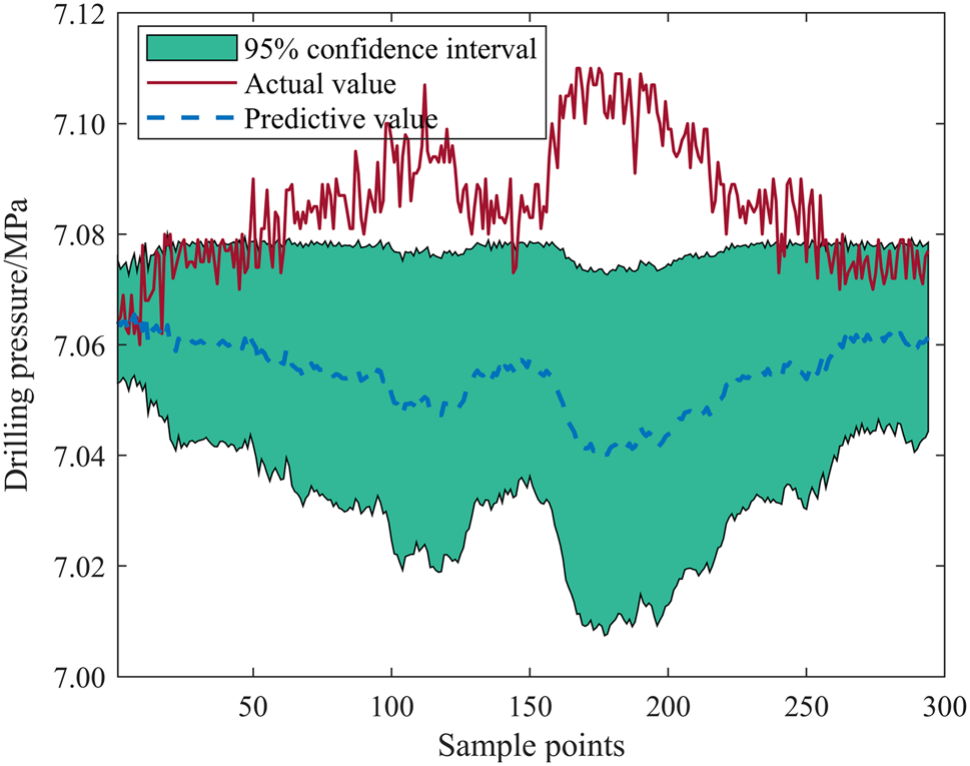
Test set solution results (kernel function = E, historical points = 6).

We employed the Root Mean Square Error (RMSE) as our evaluation metric. The optimal combination of kernel functions and historical points was found to be the RQ kernel function with 18 historical points, yielding an RMSE value of 0.0047246. The solution curve for this test set is depicted in Fig. 1. Conversely, the least effective combination was the E kernel function with 6 historical points, which produced an RMSE of 0.035704, as shown in Fig. 2.

Comparison of Figs. 1 and 2 reveals considerable variation in calculation results based on the combination of kernel functions and historical points when using the Gaussian process time series algorithm to predict bolt support drilling pressure. The best combination allows for more accurate prediction of drilling pressure trends and a narrower confidence interval, while the worst combination's predicted values diverge significantly from the actual values, offering a wide confidence interval and results with little to no referential value. Thus, it is crucial to select the relatively optimal kernel function and number of historical points before employing the Gaussian process time series algorithm for drilling pressure prediction.

In this section, we used a brute-force approach to compare and select the most effective scheme from the 160 available. While this method provided accurate results, it was time-consuming, requiring approximately 1200 s for the comparative calculation test. Therefore, we investigated the use of an optimization algorithm for iterative optimization of kernel functions and historical points.

Most optimization algorithms, however, necessitate a high number of iterations to achieve accurate optimization goals, making them more suitable for large sample optimization cases. Given our relatively small sample size of kernel function and historical point combinations, and the need to limit the number of iterations due to computation time constraints, identifying an optimization function that aligns with these characteristics is also critical.

### Hybrid optimization algorithm screening key parameters

In the process of screening key parameters for our Gaussian Process Regression model, the selection of optimization algorithms plays a pivotal role. We chose Genetic Algorithm (GA), Particle Swarm Optimization (PSO), and Ant Colony Algorithm (ACA) due to their distinct capabilities in navigating complex optimization landscapes. GA is known for its robustness and ability to explore a wide range of solutions, making it ideal for avoiding local optima. PSO is selected for its efficiency and effectiveness in finding optimal solutions quickly, which is crucial for our model's performance. Lastly, ACA is utilized for its unique approach in solving optimization problems, emulating the foraging behavior of ants, which is highly effective in complex scenarios. The amalgamation of these three algorithms allows for a more comprehensive and efficient optimization process, enhancing the accuracy of our model.

In this study, we construct GA-GPR, PSO-GPR, and ACA-GPR hybrid optimization algorithms by combining Genetic Algorithm, Particle Swarm Optimization, Ant Colony Algorithm, and Gaussian Process Regression algorithm. Before operation, a GPR conversion function is constructed with kernel function and historical points as input variables and RMSE as output dependent variables, as shown in Eq. (18).

18 $$f_{{{\text{gpr}}}} (h,i) = {\text{RMSE}}_{{{\text{gpr}}}}$$

Here, $h$ represents the code of the kernel function, with 1–10 corresponding to 10 types of kernel functions respectively, and $i$ represents the number of historical points (ranging from 5 to 20). Drilling pressure test data (Supplementary Information 1) from a roadway excavation process, drilled to a depth of 1000 mm, is used as the research sample. We use a total of 1000 sample groups, 700 for the training set, and 300 for the test set. The output ${\text{RMSE}}_{{{\text{gpr}}}}$ is the RMSE evaluation value of the test set corresponding to the Gaussian process time series regression. The ideal end goal of optimization is a test set RMSE with a kernel function = RQ and historical points = 18, i.e., 0.0047246.

All optimization functions aim for the extreme value of Eq. (18) as the optimization objective, searching for the optimal combination from 160 combinations of 10 kernel functions and 16 historical points. To highlight the application features of small samples and fewer iterations, all hybrid optimization functions are optimized with a population of 5 and 15 iterations, significantly reducing the total computation amount. While this setting may not converge within a limited number of iterations, it offers a more intuitive comparison of iterative results.

### GA-GPR algorithm

The Genetic Algorithm (GA) is an evolutionary computation method that adheres to the principle of "natural selection and survival of the fittest" seen in biology. First proposed by Professor J. Holland of the University of Michigan in 1967, it is an efficient approach for solving optimization problems^26^. In recent years, genetic algorithms have found applications in a variety of fields such as materials^27^, mathematics^28^, and medicine^29^.

The genetic algorithm operates in three key steps: (1) Selection, where superior individuals from the current population are chosen. That is, individuals with higher fitness are more likely to contribute offspring to the next generation. (2) Crossover, where a new generation of individuals is created through crossover operations with a certain probability, embodying the concept of information exchange. (3) Mutation, where the value of a string in the string-structured data is randomly altered with a certain probability.

The Genetic Algorithm (GA) operates based on the principles of natural selection and genetic inheritance. Its mechanism of selection, crossover, and mutation effectively explores the solution space, making it particularly suitable for complex optimization problems like ours. GA's ability to avoid local optima and find global solutions enhances the robustness of our GPR model optimization.

**Figure 3:**
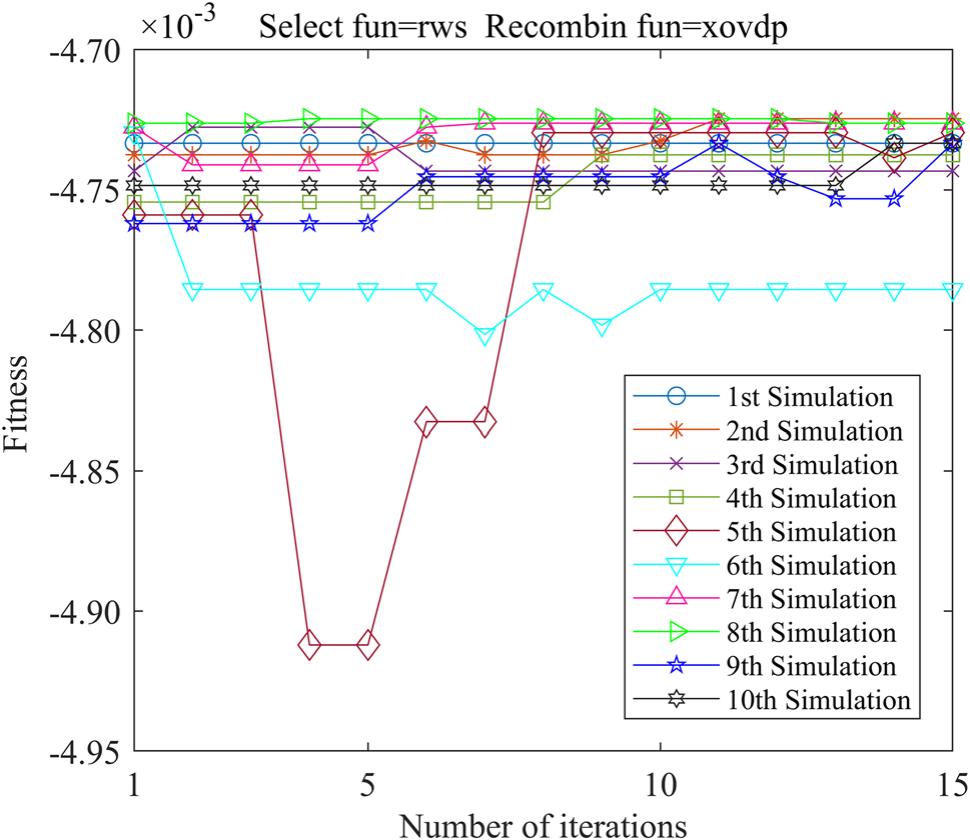
GA-GPR iteration curve (Select fun = rws Recombin fun = xovdp).

**Figure 4:**
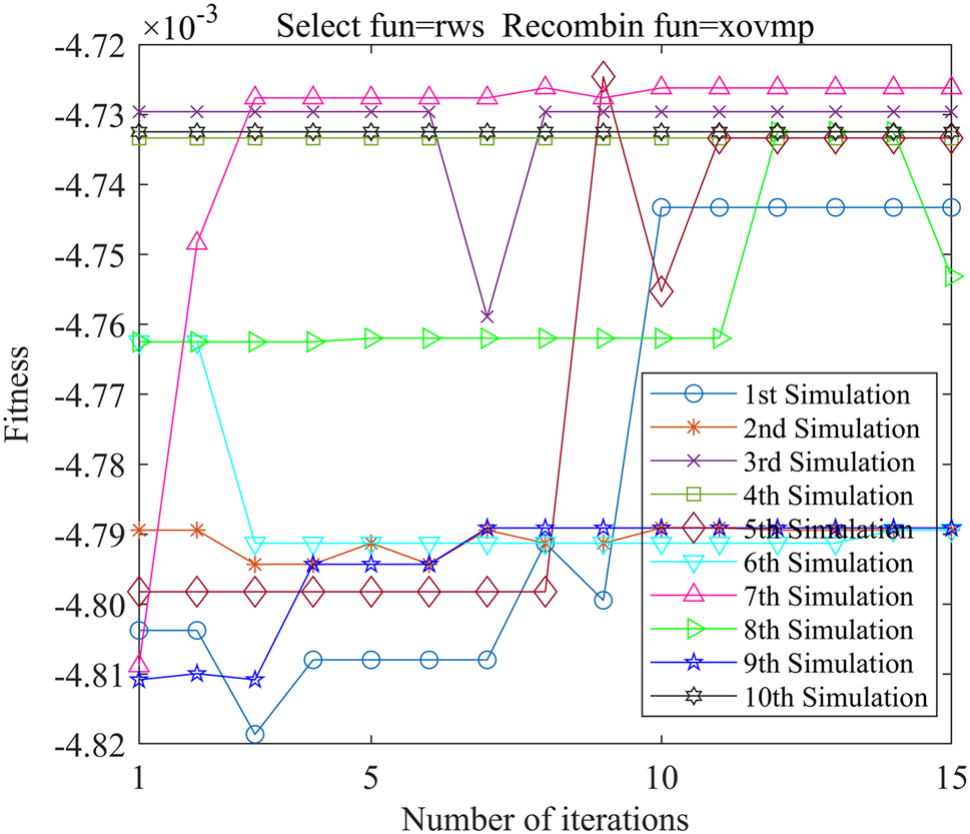
GA-GPR iteration curve (Select fun = rws Recombin fun = xovmp).

**Figure 5:**
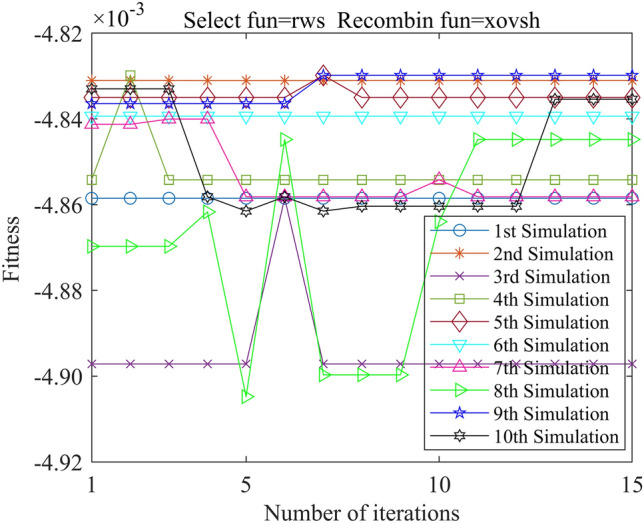
GA-GPR iteration curve (Select fun = rws Recombin fun = xovsh).

**Figure 6:**
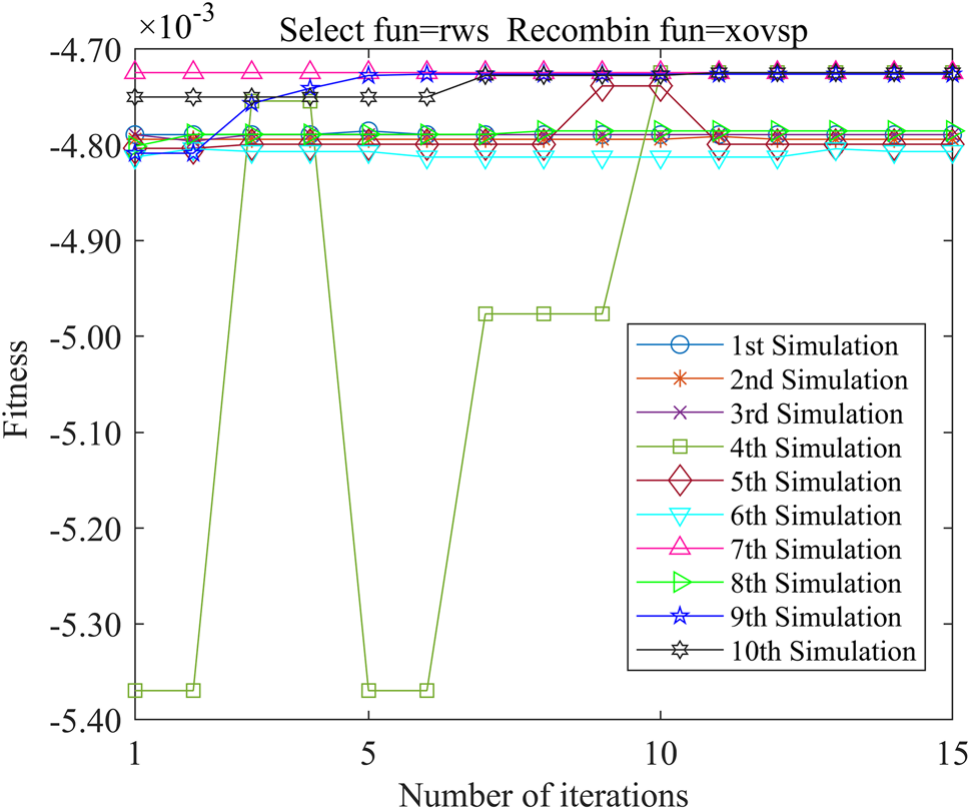
GA-GPR iteration curve (Select fun = rws Recombin fun = xovsp).

**Figure 7:**
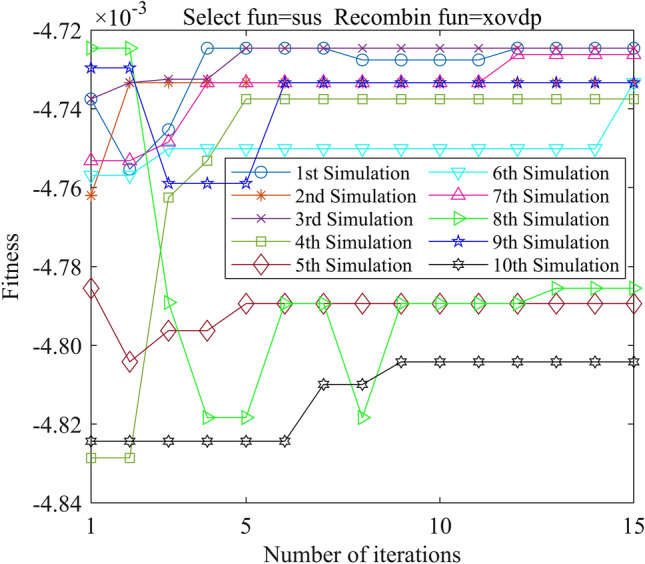
GA-GPR iteration curve (Select fun = sus Recombin fun = xovdp).

**Figure 8:**
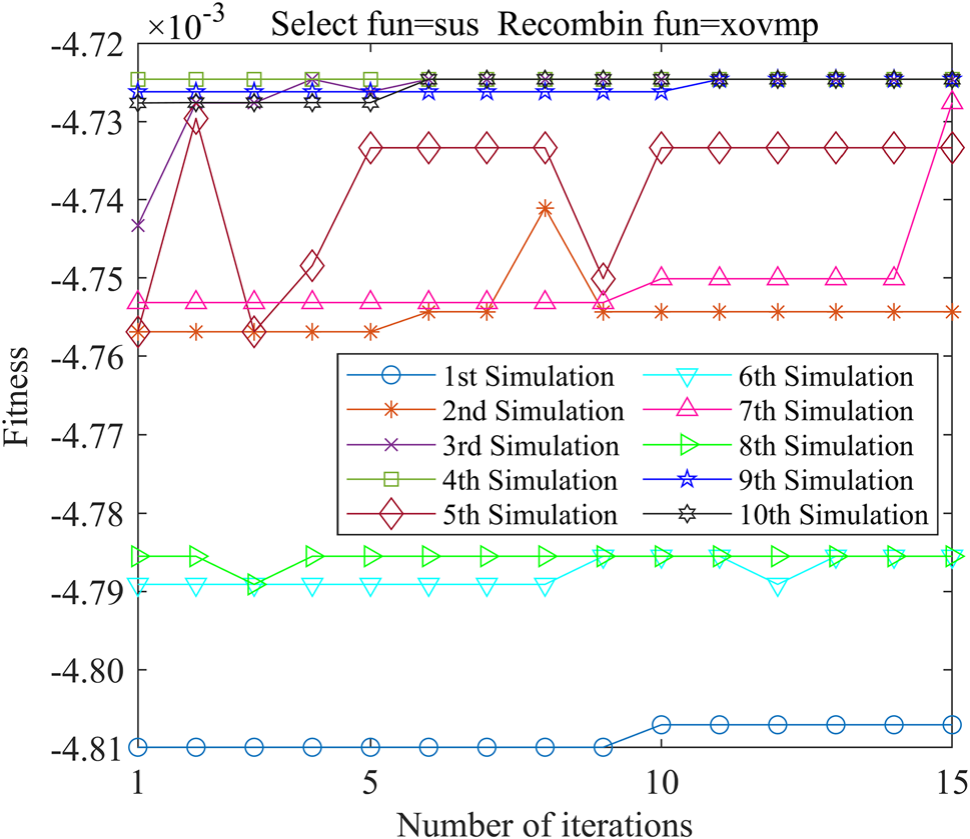
GA-GPR iteration curve (Select fun = sus Recombin fun = xovmp).

**Figure 9:**
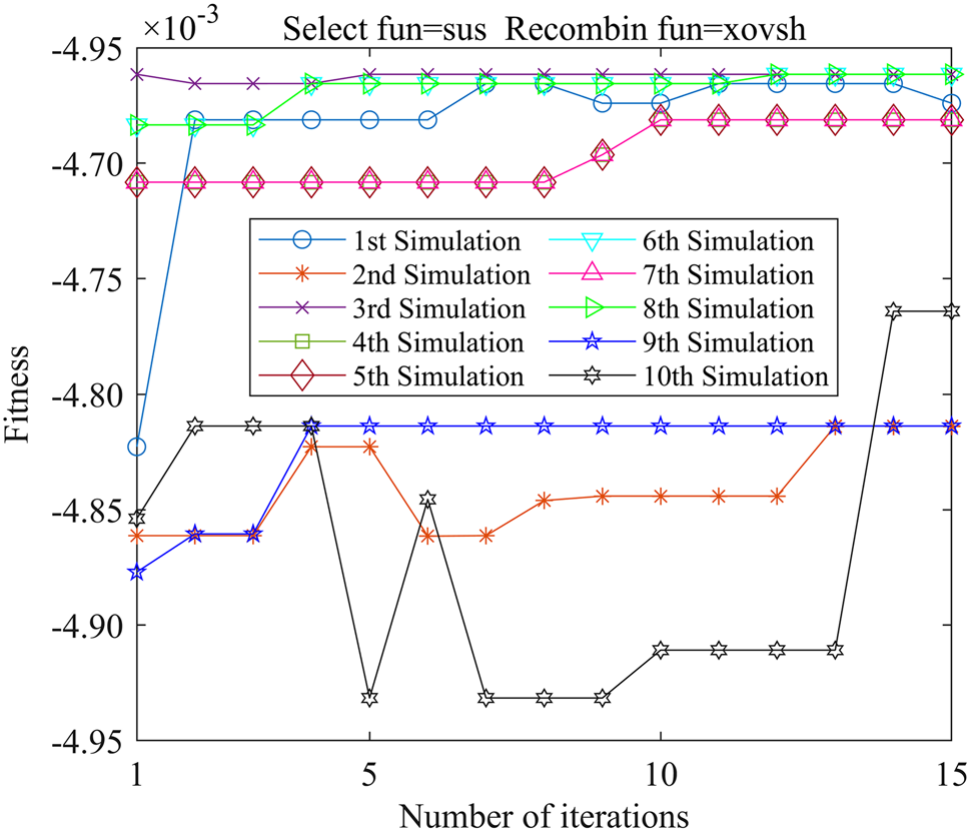
GA-GPR iteration curve (Select fun = sus Recombin fun = xovsh).

**Figure 10:**
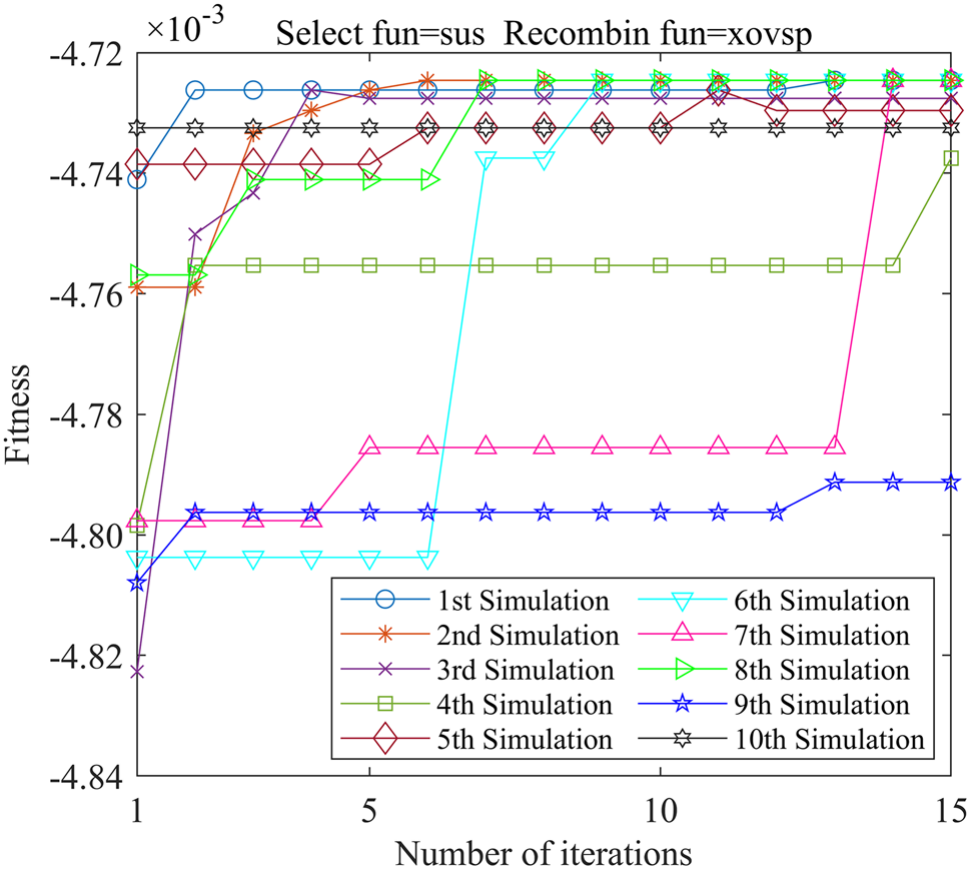
GA-GPR iteration curve (Select fun = sus Recombin fun = xovsp).

In this study, we employed the Genetic Algorithm Toolbox (gatbax) from the University of Sheffield for optimization simulation. We set the selection probability to 0.80, the crossover probability to 0.75, and the mutation probability to 0.02. The selection function includes both roulette wheel selection (rws) and stochastic universal sampling (sus), and there are four types of crossover functions: two-point crossover (xovdp), single-point crossover (xovsp), shuffle crossover (xovsh), and multi-point crossover (xovmp). In total, we have eight combination choices for the crossover functions. Each function combination was solved 10 times. Since the gatbax toolbox optimizes for maximum value and the optimization objective of formula (18) is minimum value, the optimization fitness function of GA-GPR takes a negative value for formula (18). The final iteration results are presented in Figs. 3, 4, 5, 6, 7, 8, 9 and 10.

The evaluation of iterative calculation accuracy suggests that all function combinations reached the target value (− 0.0047246) in fewer than 50% of the 10 GA-GPR simulations, indicating suboptimal performance. When the crossover functions are the same, the accuracy ratio when the selection function equals rws is smaller than when the selection function equals sus. The best solution was found to be the combination of selection function sus and crossover function xovsp, with an accuracy ratio of 40%.

Evaluating the GA-GPR combination with the optimal selection function (sus) and crossover function (xovsp) in terms of iteration calculation time, we found the average calculation time of 10 iterations to be 375 s. This is a reduction of 68.75% compared to the brute-force method, satisfying the requirement for reduced calculation time and demonstrating excellent performance.

### PSO-GPR algorithm

Particle Swarm Optimization (PSO) is an optimization algorithm that originated from the study of bird flocking behavior. It was first proposed by Kennedy and Eberhart in 1995^30^. The algorithm is relatively straightforward, updating an individual's position by tracking the individual's best-known position and the best-known position in the swarm^31^, causing all particles to continually gravitate towards the potential optimal solution until convergence^32^. In recent years, the PSO algorithm has been applied to areas such as power systems^33^ and task scheduling^34^.

A critical parameter in PSO is the inertia weight (W), which reflects a particle's ability to retain its previous velocity. A larger inertia weight enhances the global search ability, while a smaller one improves local search precision. Therefore, a decreasing inertia weight aligns better with the optimization characteristics. The PSO algorithm's strength lies in its fast convergence and ability to handle multi-dimensional spaces, contributing significantly to the refinement of our GPR mode.

**Figure 11:**
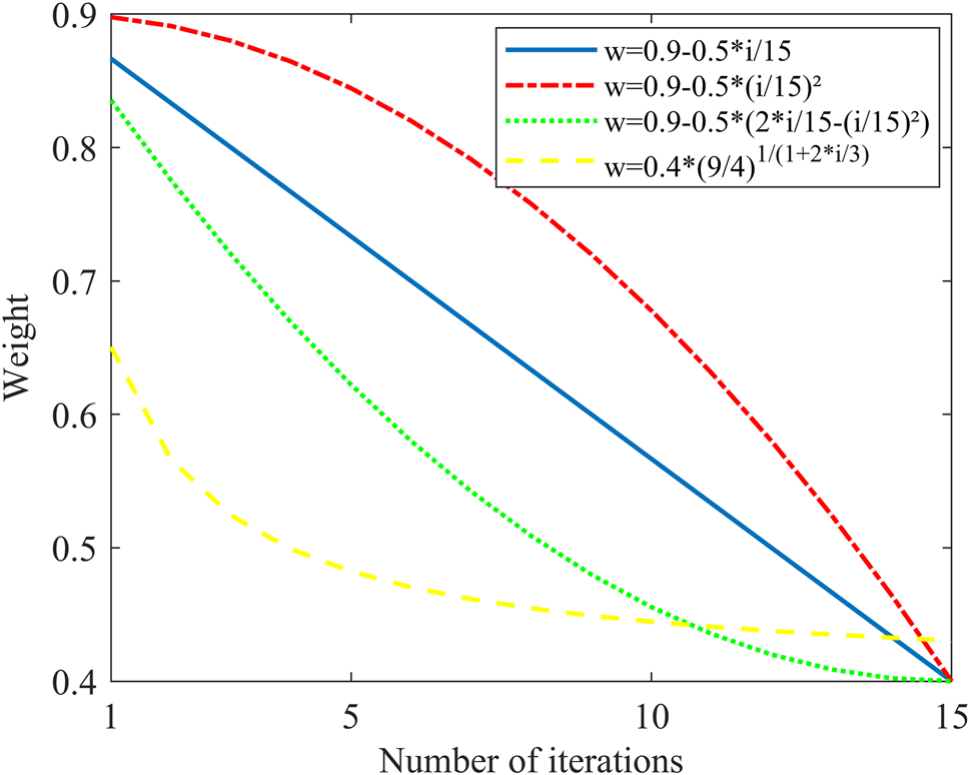
Four inertia weight reduction schemes.

**Figure 12:**
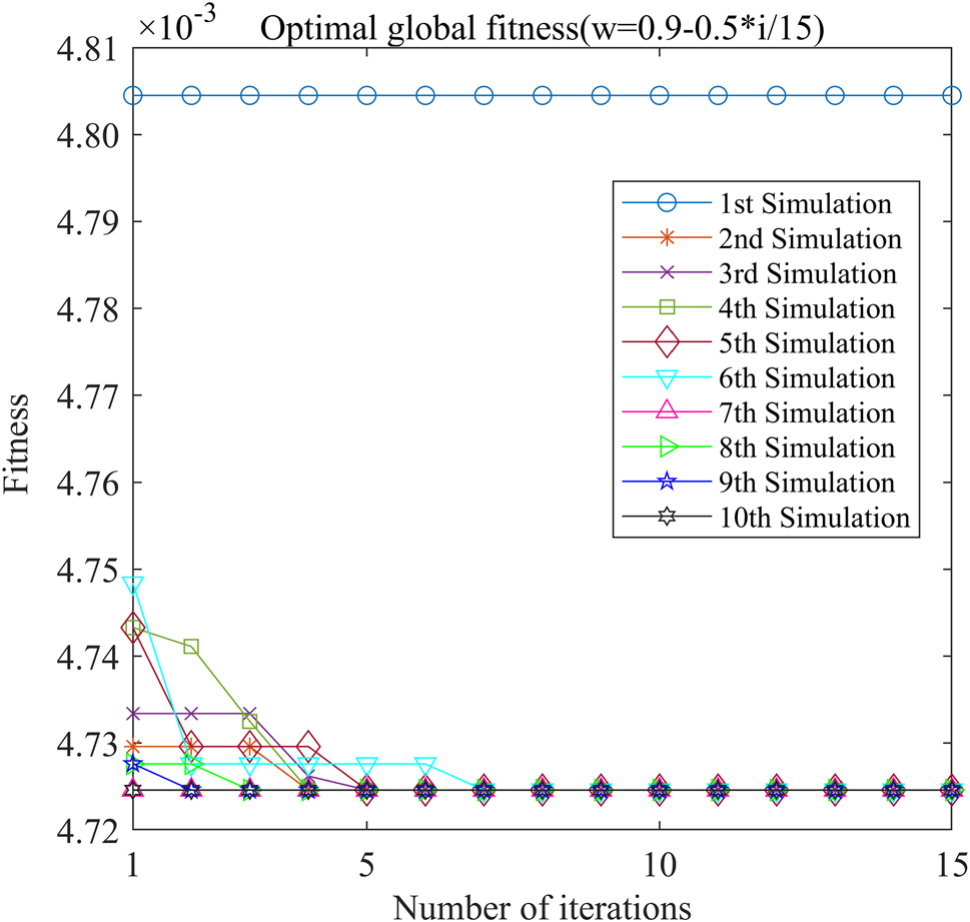
PSO-GPR iteration curve (W = 0.9–0.5*i/15).

**Figure 13:**
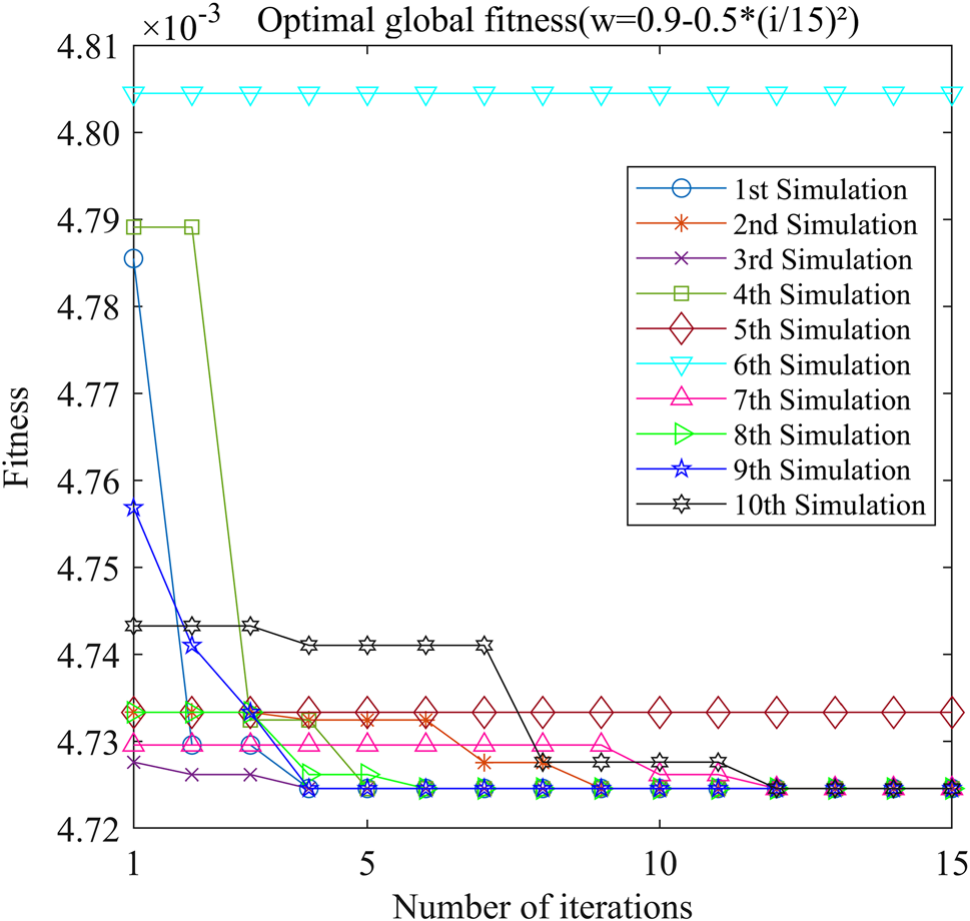
PSO-GPR iteration curve (W = 0.9–0.5*(i/15)^2^).

**Figure 14:**
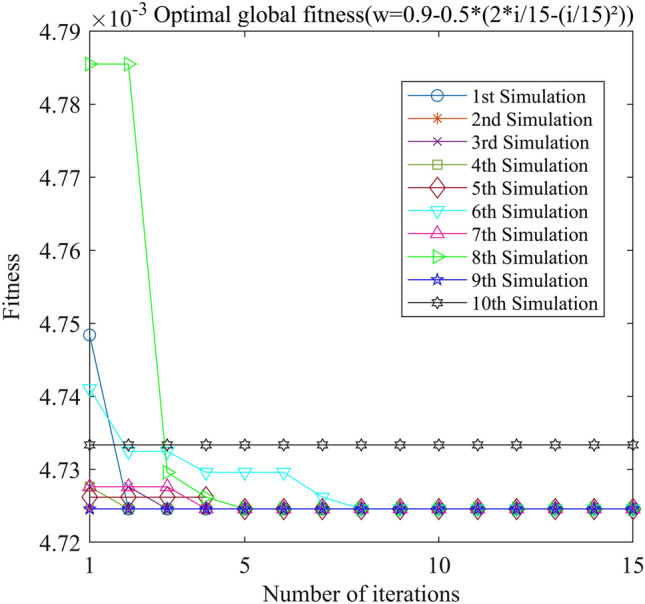
PSO-GPR iteration curve (W = 0.9–0.5*(2*i/15-(i/15)^2^)).

**Figure 15:**
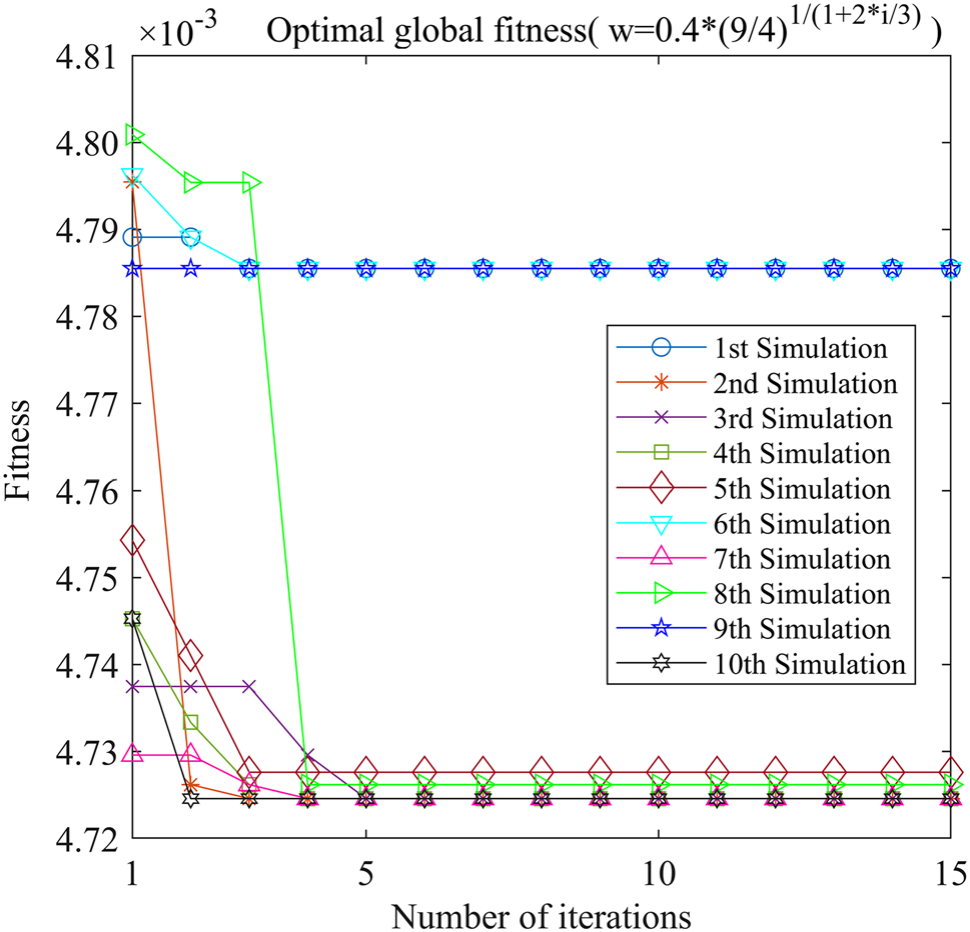
PSO-GPR iteration curve (W = 0.4*(9/4)^1/(1+2*i/3)^).

**Figure 16:**
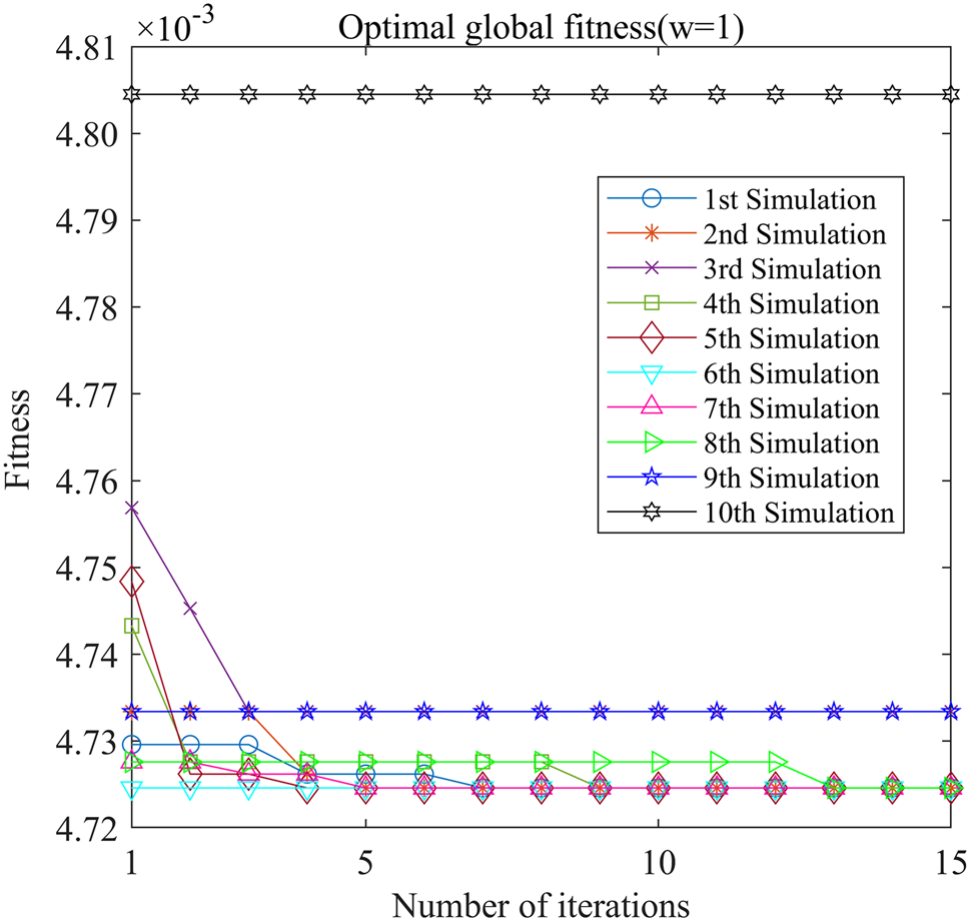
PSO-GPR iteration curve (W = 1).

In this study, we selected four classic decreasing inertia weight schemas and one schema with a constant inertia weight (W = 1), as illustrated in Fig. 11. The optimal fitness function of GA-GPR is formula (19). Moreover, the update speed range for kernel functions and historical points under the PSO-GPR solution is − 4 to 4. The individual speed update proportion parameter (C1) is 1.49445, and the swarm speed update proportion parameter (C2) is 1.49445. The final iteration results are presented in Figs. 12, 13, 14, 15 and 16.

Assessing the accuracy of iterative calculations, the ratio of reaching the target value (− 0.0047246) in the 10 PSO-GPR simulations in Figs. 12, 13 and 14 is more than 60%. The ratio for the simulations in Figs. 15 and 16 is 30%. Overall, the performance is satisfactory. The best iteration result is the PSO-GPR simulation when W = 0.9–0.5 * i/15. Under application conditions of a population size of 5 and 15 iterations, the accuracy rate reaches 80%.

In terms of iteration time, the PSO-GPR algorithm (W = 0.9–0.5*i/15) exhibits superior performance. The average calculation time across its 10 iterations is 480 s, which is 60% less than that of the brute-force method. Thus, it satisfactorily meets the requirement of reducing computation time and delivers commendable performance.

### ACA-GPR algorithm

The Ant Colony Algorithm (ACA) is an optimization methodology that emulates the foraging behavior of ants in nature^35^. It was first proposed by Marco Dorigo in his doctoral thesis in 1992^36^. As ants forage, they deposit pheromones on their paths, enabling other ants to perceive these pheromones. The concentration of pheromones represents the distance of the path, with a higher concentration indicating a shorter corresponding path^37^. Here, the pheromone can be interpreted as the fitness function value. In recent years, several scholars have proposed enhanced algorithms based on the ACA^38^, mainly applied to scheduling and route planning problems^39^.

Over time, the pheromones left by ants on the path gradually evaporate and disappear. The key parameter here is the pheromone evaporation coefficient (Rho). If Rho is too small, the previously searched path is more likely to be selected again, affecting the global search ability of the algorithm. Conversely, a large Rho can enhance global search ability but may increase many redundant search operations, thereby slowing algorithm convergence. Therefore, Rho's role is akin to inertia weight W in the PSO algorithm, and a decreasing Rho better aligns with optimization characteristics.

Ant Colony Algorithm (ACA) mimics the pheromone trail-laying and following behavior of ants, making it effective in solving discrete optimization problems. It utilizes a probabilistic technique for finding the shortest paths, which is instrumental in exploring the optimal combination of kernel function and historical points in our GPR model. ACA's unique approach to problem-solving complements the global search capabilities of GA and the rapid convergence of PSO in our hybrid optimization framework.

**Figure 17:**
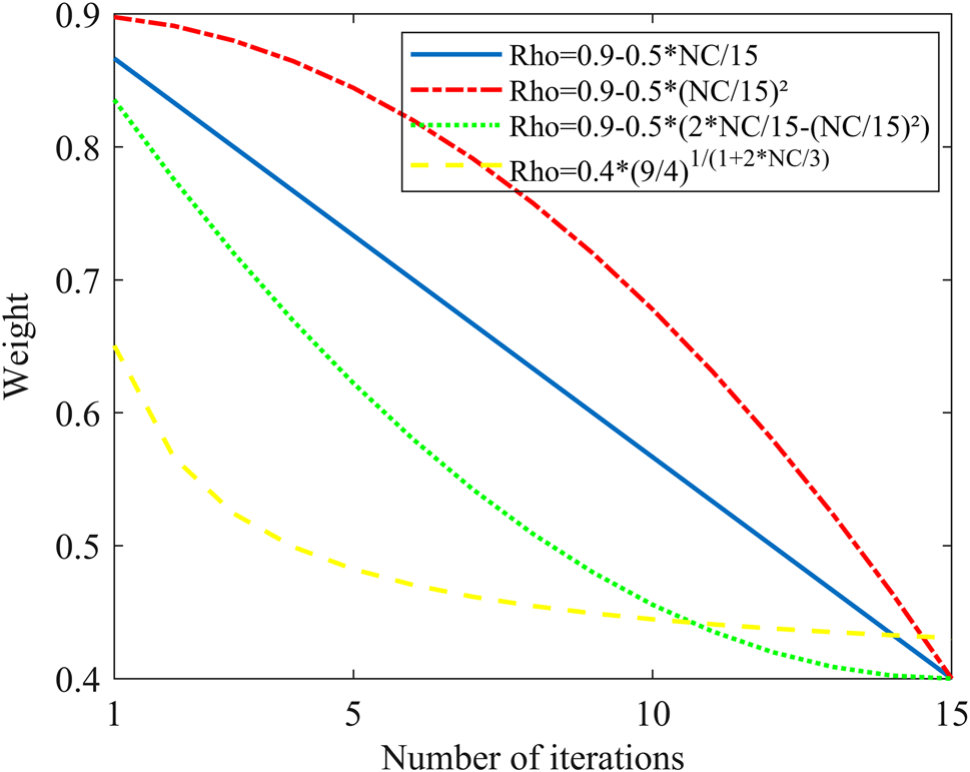
Four Rho reduction schemes.

**Figure 18:**
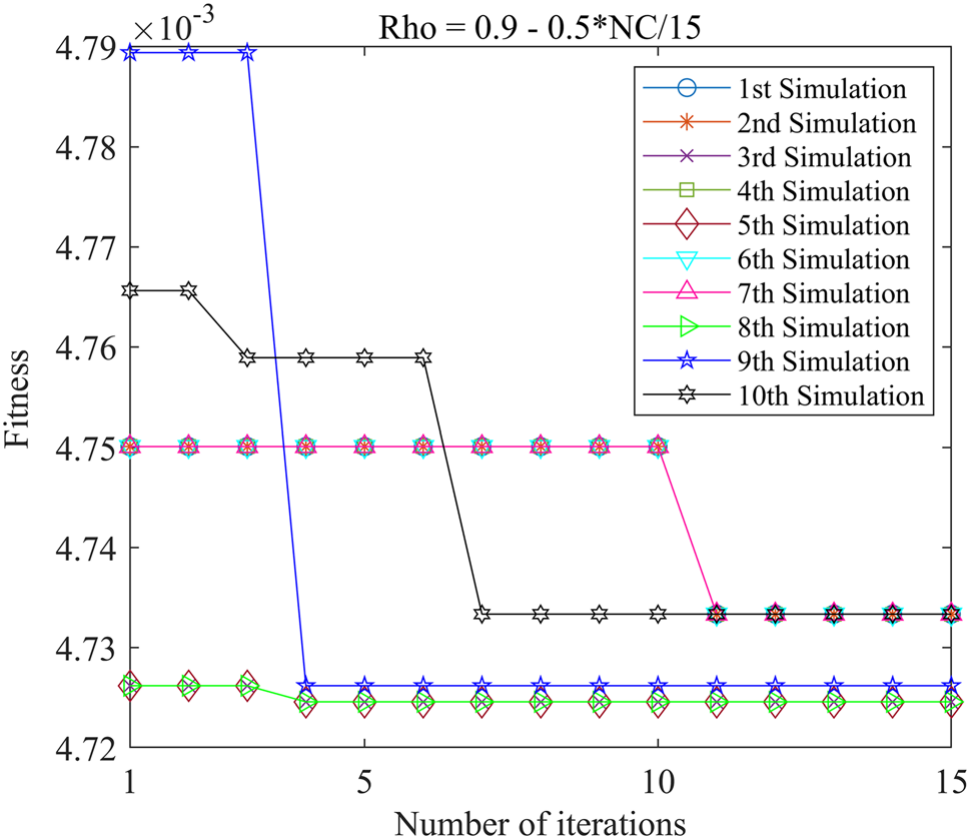
ACA-GPR iteration curve (Rho = 0.9–0.5*i/15).

**Figure 19:**
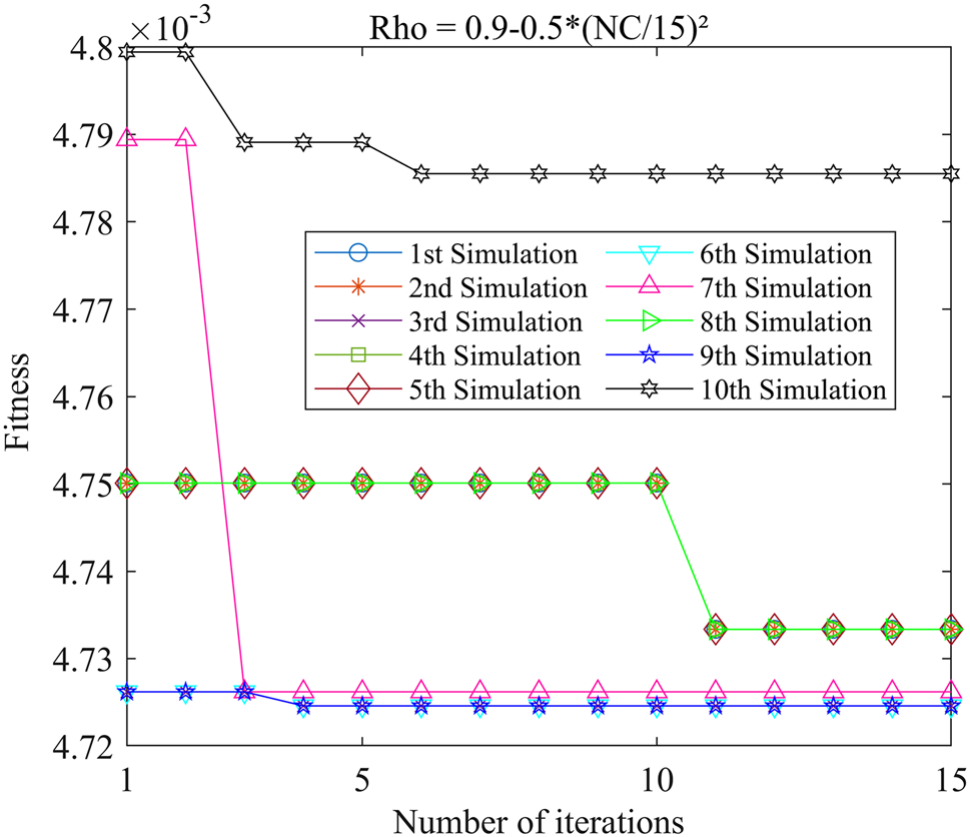
ACA-GPR iteration curve (Rho = 0.9–0.5*(i/15)^2^).

**Figure 20:**
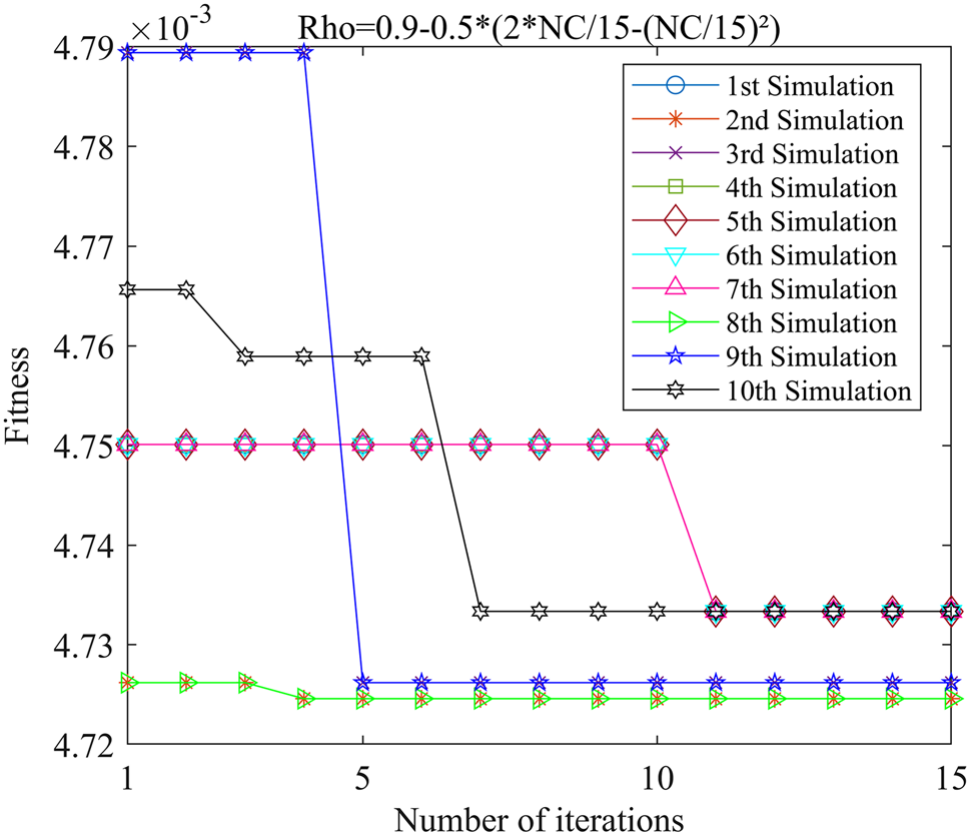
ACA-GPR iteration curve (Rho = 0.9–0.5*(2*i/15-(i/15)^2^)).

**Figure 21:**
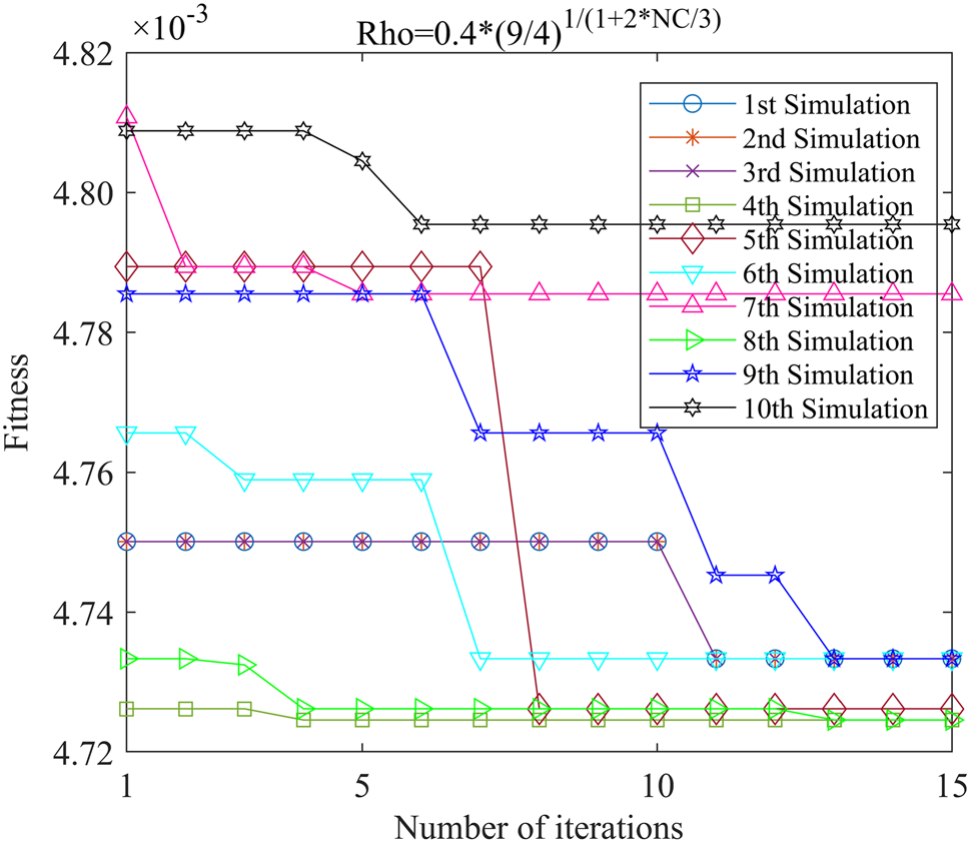
ACA-GPR iteration curve (Rho = 0.4*(9/4)^1/(1+2*i/3)^).

**Figure 22:**
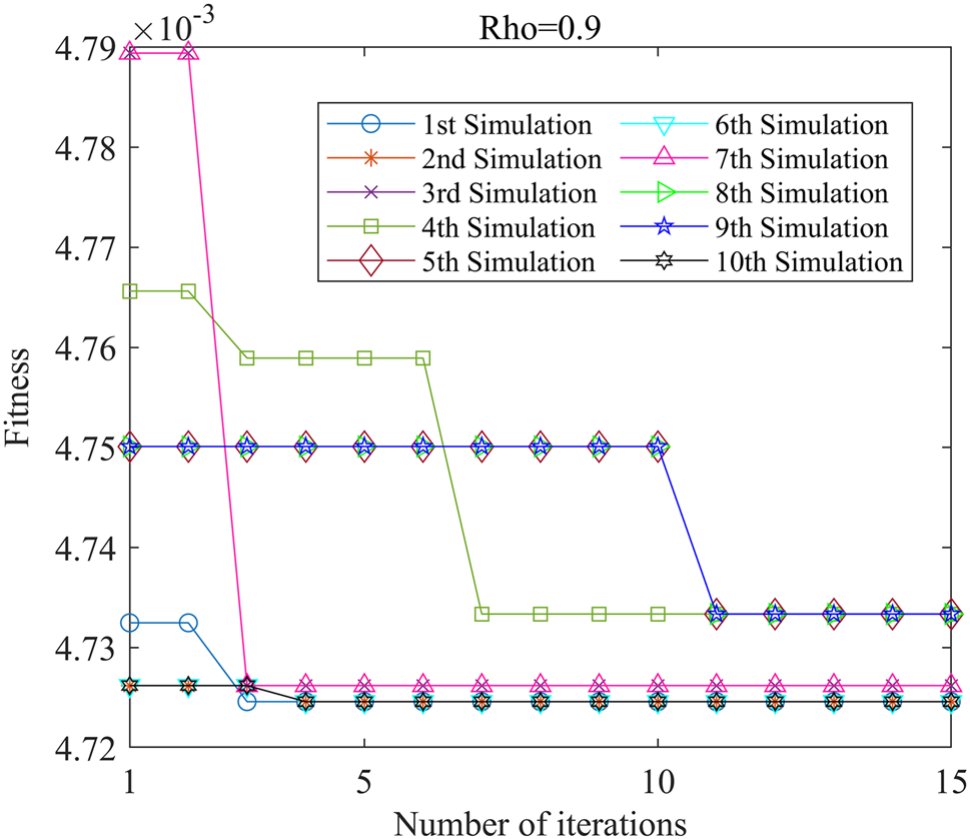
ACA-GPR iteration curve (Rho = 0.9).

In this study, we selected four classic Rho decay schemas and one schema with a constant Rho of 0.9, as depicted in Fig. 17. The optimal fitness function of ACA-GPR is also formula (19). Additionally, the transition probability constant of the ACA-GPR solution is 0.1, and the local search step is 2. The final iteration results are presented in Figs. 18, 19, 20, 21 and 22.

Assessing the accuracy of iterative calculations, the ratio of reaching the target value (− 0.0047246) in the 10 ACA-GPR simulations in Figs. 18, 19, 20 and 21 is less than or equal to 30%. The ratio for the simulation in Fig. 22 is 40%. Overall, the performance is subpar. Given limited calculation and iteration times, ACA-GPR exhibits certain shortcomings, such as slow convergence speed and a propensity to get stuck in local optima.

In terms of iteration calculation time, all ACA-GPR simulations exhibit lengthy computation times. Even for the best-performing group (Rho = 0.9), the average time for 10 iterations is 1882s, which is 56.83% longer than the brute-force method. This excess in iteration time undermines the significance of the optimization algorithm, resulting in poor performance.

## Result comparison

Taking the drilling pressure test data when drilling 1000 mm in the process of roadway excavation as the research sample, the optimization results of three hybrid optimization algorithms GA-GPR, PSO-GPR, and ACA-GPR on the relative optimal kernel function and the relative optimal historical points are different. Results show that PSO-GPR performs best, followed by GA-GPR, and lastly ACA-GPR. PSO-GPR algorithm is more suitable for screening the key parameter kernel function and historical points in the Gaussian process time series algorithm applied to the prediction of drilling pressure in bolt support. When the sample size is small and the number of iterations is small, PSO-GPR can achieve 80% accuracy with a 60% reduction in time.

In this study, the PSO-GPR model has demonstrated an 80% accuracy rate in predicting the pre-drilling pressure for bolt support. It's important to contextualize this level of accuracy within the operational framework of underground drilling. For practical drilling operations, a deviation of 1–2 MPa in predicted pressure is typically within acceptable limits, considering the use of load-sensitive hydraulic systems in modern drilling equipment. The primary objective of this prediction is not to pinpoint the exact drilling pressure, but rather to enable proactive adjustments, such as reducing drilling speed in advance to protect the drill bit and optimize drilling efficiency. This preemptive adjustment approach, facilitated by our PSO-GPR model, enhances the safety and efficiency of the drilling process by mitigating risks associated with unexpected pressure spikes and equipment stress. Thus, while the model's 80% prediction accuracy might not capture every nuance of the underground environment, it provides a significant operational advantage by allowing for early interventions that maintain equipment integrity and support safety protocols.

In previous studies, the hybrid optimization algorithm is comprehensively evaluated by iterative calculation accuracy and iterative calculation time. Therefore, we introduce the evaluation function of the hybrid optimization algorithm to quantitativelyevaluate the hybrid optimization algorithm. This is shown by Eq. (19):

19 $$EF = \left\{ {\begin{array}{*{20}l} {C + T} \hfill & {T \ge 0} \hfill \\ 0 \hfill & {T < 0} \hfill \\ \end{array} } \right.$$

In the formula, ‘*C*’ signifies the accuracy rate of iterative calculations, and ‘*T*’ stands for the reduction rate in computation time compared to the brute-force method. ‘*EF*’ denotes the evaluation index of the hybrid algorithm, with a higher value indicating superior algorithm performance.

## Application of predicting drilling pressure for anchor rod support

### Methodology

**Figure 23:**
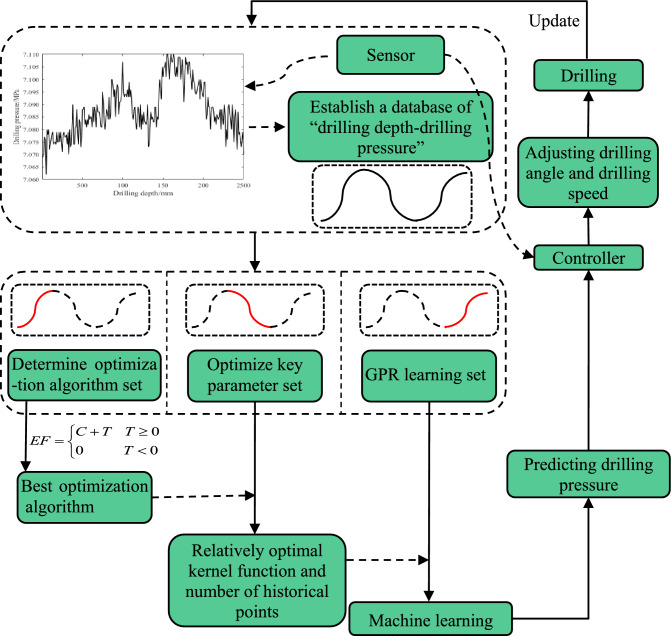
Application flowchart.

Building on previous research, we have established a predictive system for drilling pressure in underground bolt support. The basic steps are as follows:

The application flow chart is shown in Fig. 23.

**Step 1:** Pressure and displacement sensors are outfitted on the bolt support drill to monitor varying drilling pressures at different depths. Over time, this data is accumulated to form a "drilling depth - drilling pressure" database.

**Step 2:** The database is partitioned into three sets: the "Optimization Algorithm Determination Set," the "Key Parameter Optimization Set," and the "GPR Learning Set".

**Step 3:** Using the "Optimization Algorithm Determination Set" as a sample, a quantitative evaluation is conducted using the evaluation function of the hybrid optimization algorithm, allowing the selection of the best optimization algorithm for this data type. The quality of the optimization algorithm is related to the law of data type, which remains constant once determined.

**Step 4:** Utilizing the "Key Parameter Optimization Set" as a sample, the optimal optimization algorithm is used to select the optimal kernel function and number of historical points for Gaussian Process time series regression of bolt support drilling pressure. This step needs to be performed regularly, updating the optimal parameters.

**Step 5:** The "GPR Learning Set" serves as the sample for machine learning, utilizing the currently optimal kernel function and number of historical points as parameters.

**Step 6:** Predict the drilling pressure of the subsequent bolt support, and use the predicted pressure value to automatically set the relevant drilling angle, drilling speed, and other parameters. Simultaneously, the actual drilling pressure monitored by the pressure sensor is stored, and the "GPR Learning Set" and the "drilling depth - drilling pressure" database are continuously updated.

### Case study

Data for this case study was collected from the feed pressure of the electro-hydraulic control anchor drilling frame used by CCTEG Taiyuan Research Institute Co., Ltd in Shaanxi Huangling No. 2 Coal Mine Co., Ltd. In the rapid excavation system designed and developed by CCTEG Taiyuan Research Institute Co., Ltd., the drill frame can perform one-key automatic drilling, equipped with a displacement sensor and a pressure sensor to monitor and store the corresponding pressure value of the drill frame at different depths in real-time.

The drilling pressure test data at 1000 mm (Supplementary Information 1), 1200 mm (Supplementary Information 2), 2400 mm (Supplementary Information 3), and 3000 mm (Supplementary Information 4) depths during roadway excavation were used for the "Optimization Algorithm Determination Set", the "Key Parameter Optimization Set", and the "GPR Learning Set". The last 300 drilling pressures at 2400 mm and 3000 mm were predicted using the algorithm.

According to the studies, the PSO-GPR (w = 0.9–0.5*i/15) was found to be the best hybrid optimization algorithm for the current data type, as determined through the "Optimization Algorithm Determination Set".

**Figure 24:**
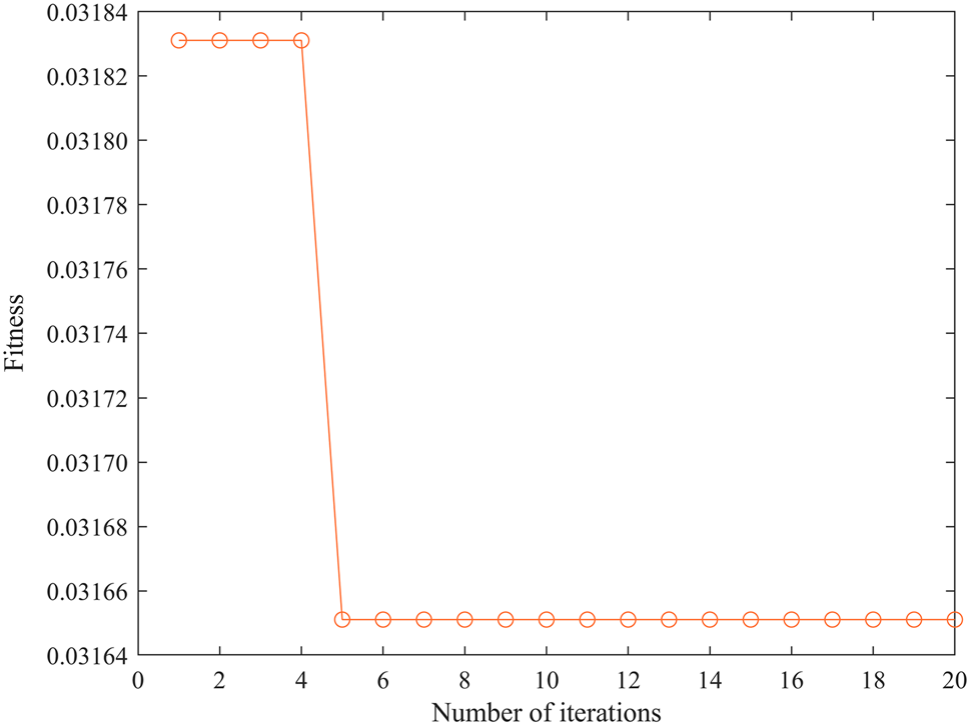
Iterative optimization curve.

Using the "Key Parameter Optimization Set" as input data, the optimal parameters of Gaussian Process time series regression were determined by the PSO-GPR algorithm. The algorithm population number of PSO-GPR (w = 0.9–0.5*i/15) was set to 8, the iteration number to 20, and the remaining parameters were left unchanged. The iterative optimization curve is shown in Fig. 24.

The total time for 20 iterations was 210 s, the optimization result was 0.0316511, the corresponding kernel function was Matern5/2, and the number of historical points was 9.

**Figure 25:**
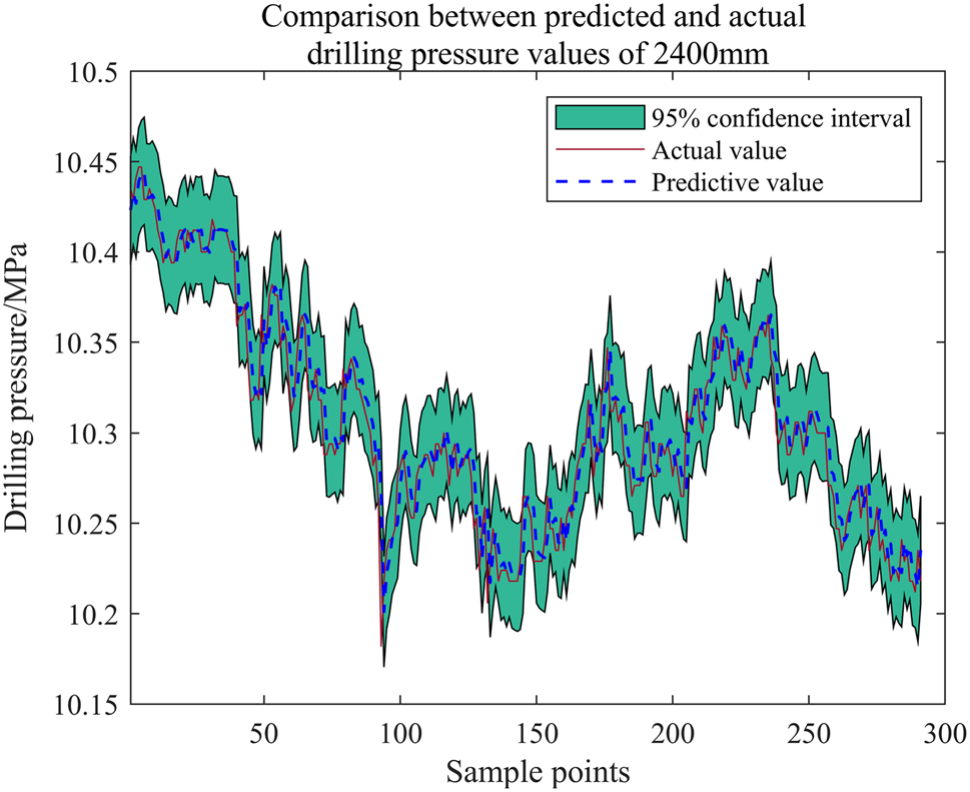
Test set solution results (2400 mm).

**Figure 26:**
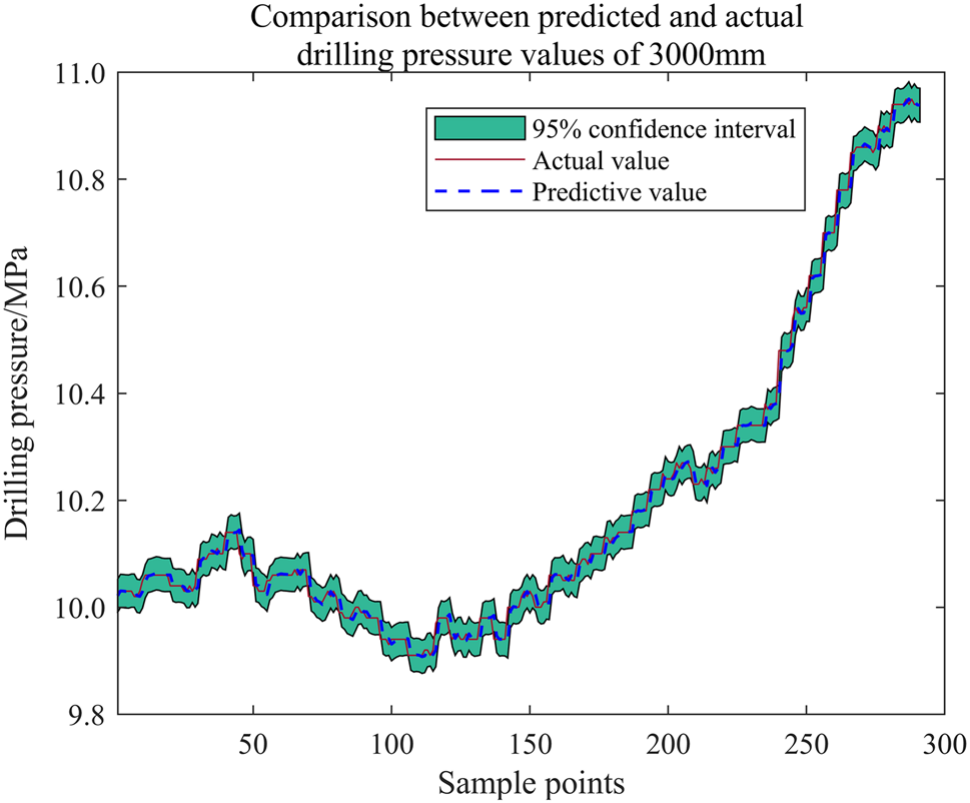
Test set solution results (3000 mm).

Using kernel function = Matern5/2 and historical points = 9, the "GPR Learning Set" was learned, and the subsequent drilling pressures were predicted. The test set prediction results for 2400 mm and 3000 mm drilling data are shown in Figs. 25 and 26 respectively.

Figures 25 and 26 reveal excellent prediction results, with a narrow confidence interval bandwidth. The drilling pressure prediction method for bolt support, based on the hybrid optimization algorithm to screen the key parameters of Gaussian process time series regression, exhibits a certain level of generalization.

## Methods

In this study, we developed a robust methodology to optimize the Gaussian Process Regression (GPR) model for predicting drilling pressure in underground bolt support, integrating various hybrid algorithms for enhanced accuracy. Central to our optimization approach is the implementation of a fitness function and the application of three distinct optimization algorithms.

### Fitness function (OptimizeGPR function)

The OptimizeGPR function, based on Eq. (18), computes the RMSE as the fitness indicator for the GPR model. It processes the data with historical points and kernel function types, undergoing normalization and a 70:30 training–testing split. The function is designed to identify the parameter combination that minimizes RMSE across different kernel configurations.

### GA-GPR algorithm

**Figure 27:**
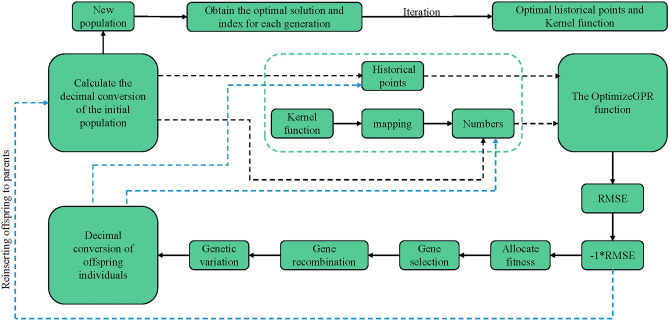
GA-GPR algorithm flowchart.

Utilizing the Genetic Algorithm Toolbox (gatbax) from the University of Sheffield, this algorithm involves genetic operations like selection, crossover, and mutation, targeting the maximization of the fitness value by operating on the negative value of the OptimizeGPR function. The algorithm iteratively evolves solutions, focusing on minimizing the RMSE to identify the optimal historical points and kernel function types. The process is detailed in Fig. 27.

### PSO-GPR algorithm

**Figure 28:**
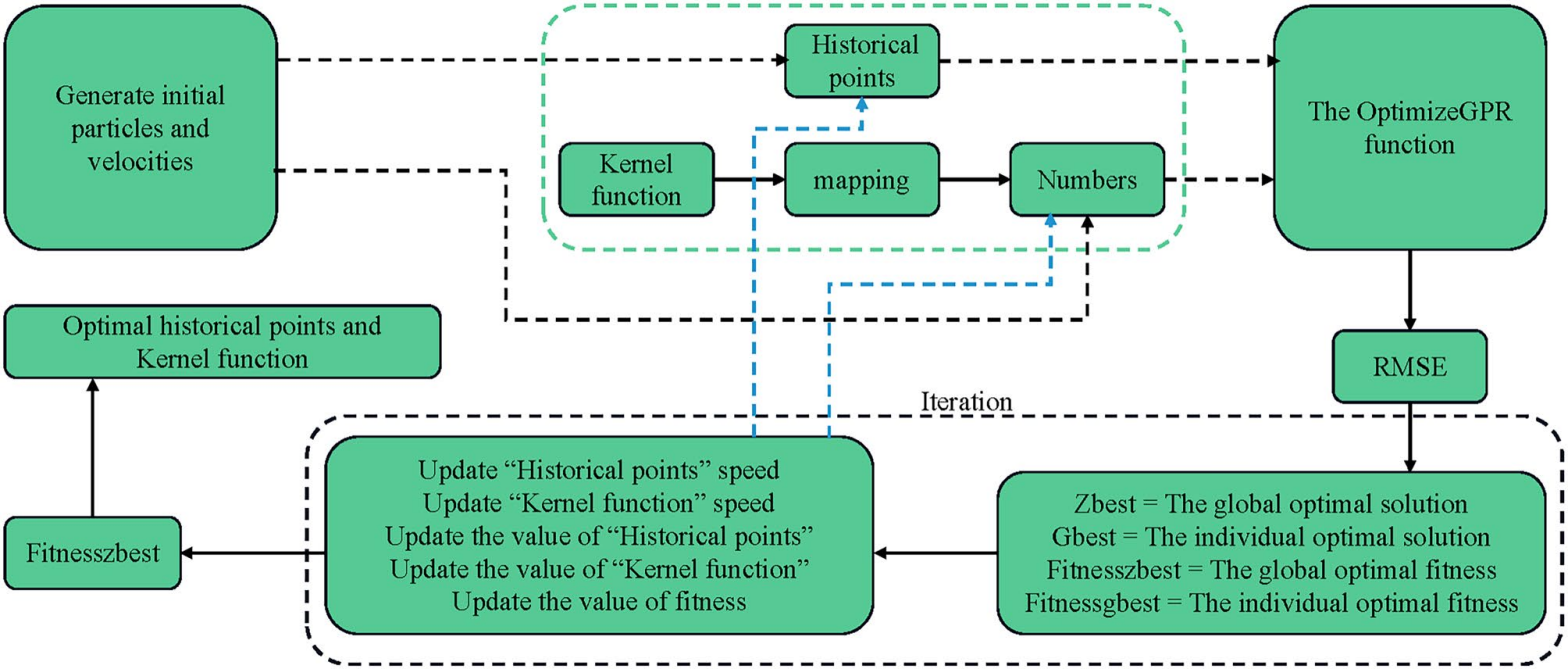
PSO-GPR algorithm flowchart.

The PSO-GPR algorithm is built upon the principles of particle swarm optimization, where particles' positions and velocities are dynamically adjusted according to their fitness, evaluated by the OptimizeGPR function. The iterative process seeks to efficiently converge to the GPR parameters that minimize RMSE. The algorithm's dynamic adaptation is illustrated in Fig. 28.

### ACA-GPR algorithm

**Figure 29:**
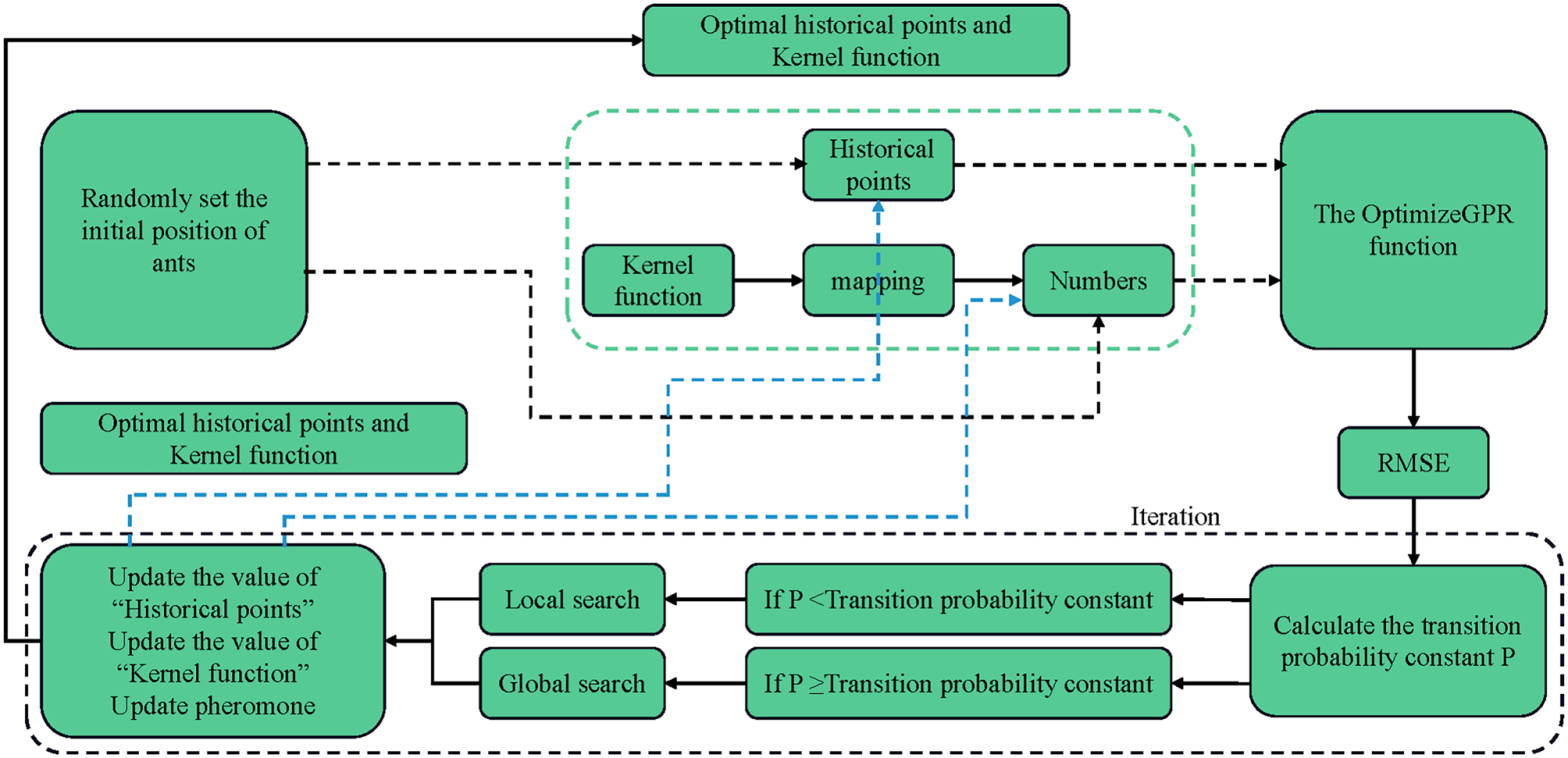
ACA-GPR algorithm flowchart.

In the ACA-GPR method, we constructed functions such as the Rho function to simulate ant colony behavior. The ants explore the solution space, guided by the OptimizeGPR function to find the most effective combination of parameters that minimize the RMSE.The algorithm's implementation is depicted in Fig. 29.

## Conclusion

This study presents a novel method for predicting bolt support drilling pressure using a hybrid optimization algorithm for Gaussian process time series regression. Unlike traditional pre-drilling pressure prediction methods, such as seismic interval velocity, our approach introduces the innovative application of machine learning. Prior techniques primarily focused on material properties and did not adapt well to varying geological conditions. In contrast, our method demonstrates enhanced prediction accuracy and versatility, marking a significant leap in underground drilling practices.

The key conclusions are:

1. Gaussian process time series regression, depending on the selection of kernel function and historical points, effectively predicts drilling pressure within a confidence interval. The optimal combination significantly enhances prediction accuracy and narrows the confidence interval.
2. Among the evaluated hybrid optimization algorithms (GA-GPR, PSO-GPR, ACA-GPR), PSO-GPR demonstrates superior performance, achieving 80% prediction accuracy with substantial computational efficiency, including a 60% reduction in computation time. This level of accuracy is deemed sufficient for practical applications in underground drilling operations, considering the typical tolerances and operational requirements.
3. The established prediction system for underground bolt support proves its generalizability across various test data, highlighting potential improvements in operational efficiency and safety. The 80% accuracy rate of the PSO-GPR model, while not perfect, is adequate for enabling proactive adjustments in drilling operations, such as optimizing drilling speed and angle to enhance equipment safety and drilling efficiency.

### Limitations

While the study presents significant advancements, it's important to acknowledge certain limitations. The accuracy of the model, while generally sufficient for operational needs, may not capture all nuances of complex underground environments. Additionally, the model's performance is subject to the quality of available data and may require adjustments when applied to different geological conditions. Future research could focus on enhancing the model's adaptability and testing it in a wider range of scenarios.

### Supplementary Information

Supplementary Information 1.

Supplementary Information 2.

Supplementary Information 3.

Supplementary Information 4.

## Data Availability

The time-series data of drilling pressure for anchor rod support at various depths used in this study has been deposited in Zenodo and can be accessed directly via the following doi: 10.5281/zenodo.8396258. The data includes measurements at drilling depths of 1000 mm, 1200 mm, 2400 mm, and 3000 mm. The source code for the three hybrid optimization Gaussian Process Regression (GPR) algorithms used in this study, including Genetic Algorithm-GPR (GA-GPR), Particle Swarm Optimization-GPR (PSO-GPR), and Ant Colony Algorithm-GPR (ACA-GPR), has also been deposited in Zenodo. This code is essential for understanding the methodology used for optimizing the prediction of drilling pressure in bolt support systems. It can be accessed directly via the following doi: 10.5281/zenodo.10429027.
